# Genome-wide DNA methylation levels and altered cortisol stress reactivity following childhood trauma in humans

**DOI:** 10.1038/ncomms10967

**Published:** 2016-03-21

**Authors:** Lotte C. Houtepen, Christiaan H. Vinkers, Tania Carrillo-Roa, Marieke Hiemstra, Pol A. van Lier, Wim Meeus, Susan Branje, Christine M. Heim, Charles B. Nemeroff, Jonathan Mill, Leonard C. Schalkwyk, Menno P. Creyghton, René S. Kahn, Marian Joëls, Elisabeth B. Binder, Marco P. M. Boks

**Affiliations:** 1 Department of Psychiatry, Brain Center Rudolf Magnus, University Medical Center Utrecht, Utrecht 3584 CX, The Netherlands; 2 Department of Translational Research in Psychiatry, Max Planck Institute of Psychiatry, Munich 80804, Germany; 3 Research Centre Adolescent Development, Department Youth & Family, University Utrecht (UU), Utrecht 3584 CS, The Netherlands; 4 Department of Developmental Psychology, VU University, Amsterdam 1081 BT, The Netherlands; 5 Department of Developmental Psychology, Tilburg University 5000 LE, Tilburg, The Netherlands; 6 Institute of Medical Psychology, Charité-University Medicine, Medical Centre, 10117 Berlin, Germany; 7 Department of Biobehavioral Health, Pennsylvania State University, University Park, PA 16802, USA; 8 Department of Psychiatry and Behavioral Sciences, Leonard M. Miller School of Medicine, University of Miami, Miami, 33136, Florida, USA; 9 University of Exeter Medical School, University of Exeter, EX2 5DW, Devon, UK; 10 Institute of Psychiatry, Psychology & Neuroscience, King's College London, SE5 8AF, London, UK; 11 School of Biological Sciences, University of Essex, CO4 3SQ Colchester, UK; 12 Hubrecht Institute-KNAW and University Medical Center Utrecht (UMCU), Utrecht 3584CT, The Netherlands; 13 Department of Translational Neuroscience, Brain Center Rudolf Magnus, University Medical Center Utrecht (UMCU), Utrecht 3584 CG, The Netherlands; 14 Department of Psychiatry and Behavioral Sciences, Emory University School of Medicine, Atlanta, Georgia 30329, USA

## Abstract

DNA methylation likely plays a role in the regulation of human stress reactivity. Here we show that in a genome-wide analysis of blood DNA methylation in 85 healthy individuals, a locus in the Kit ligand gene (*KITLG*; cg27512205) showed the strongest association with cortisol stress reactivity (*P*=5.8 × 10^−6^). Replication was obtained in two independent samples using either blood (*N*=45, *P*=0.001) or buccal cells (*N*=255, *P*=0.004). *KITLG* methylation strongly mediates the relationship between childhood trauma and cortisol stress reactivity in the discovery sample (32% mediation). Its genomic location, a CpG island shore within an H3K27ac enhancer mark, and the correlation between methylation in the blood and prefrontal cortex provide further evidence that *KITLG* methylation is functionally relevant for the programming of stress reactivity in the human brain. Our results extend preclinical evidence for epigenetic regulation of stress reactivity to humans and provide leads to enhance our understanding of the neurobiological pathways underlying stress vulnerability.

## Abstract (web summary)

Exposure to childhood trauma is a major risk factor for the development of almost all psychiatric disorders. By epigenome-wide studies, here, Houtepen *et al*. show that DNA methylation at a locus in the Kit ligand gene (KITLG) mediates the relationship between childhood trauma and cortisol stress reactivity.

## Graphical abstract

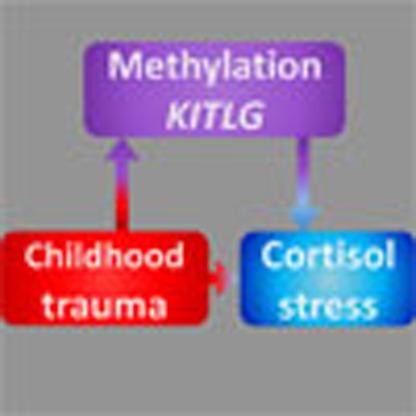

Exposure to childhood trauma is a major risk factor for the development of almost all psychiatric disorders^1^, including depression^2^, post-traumatic stress disorder (PTSD)^3^ and schizophrenia^4^. Childhood trauma is also associated with blunted or increased activity of the hypothalamic–pituitary–adrenal (HPA) axis^5^,^6^ (Supplementary Table 1 for a literature overview). These neuroendocrine changes may underlie the increased risk for psychiatric disorders across the life span. However, our understanding of how early life trauma can have such persistent detrimental effects is currently limited.

Epigenetic alterations may at least partially be involved in the lasting impact of childhood trauma. Preclinical studies have shown a consistent link between the early life environment, DNA methylation changes and adult stress reactivity and behaviour^7^,^8^. In humans, the long-term impact of traumatic stress on DNA methylation patterns is supported by several studies, which mainly focused on single genes^9^, particularly on the glucocorticoid receptor gene that is pivotal for adequate HPA-axis functionality^10^,^11^,^12^,^13^,^14^,^15^. Even though hypothesis-driven studies have convincingly demonstrated a relation between traumatic stress and DNA methylation, the persistent detrimental influence of childhood trauma is unlikely to result from epigenetic modifications in a single gene^16^. Recently, two clinical studies investigated genome-wide methylation changes associated with childhood trauma^17^ and trauma exposure in PTSD^16^, but no study has investigated functional changes in endocrine stress reactivity using an unbiased genome-wide approach.

The main aim of this study is to provide an unbiased investigation of the role of DNA methylation in cortisol stress reactivity and its relationship with childhood trauma. To this end, we perform a genome-wide DNA methylation analysis for cortisol stress reactivity in healthy individuals. We identify a locus on the *KITLG* gene (cg27512205) that is not only associated to cortisol stress reactivity, but also partly mediates the relationship between childhood trauma and cortisol stress reactivity. Furthermore, we replicate the association between cortisol stress reactivity and methylation at the *KITLG* locus in two independent samples measuring methylation in either whole blood or buccal (cross-tissue) DNA.

## Results

### DNA methylation and cortisol stress reactivity

**Figure 1:**
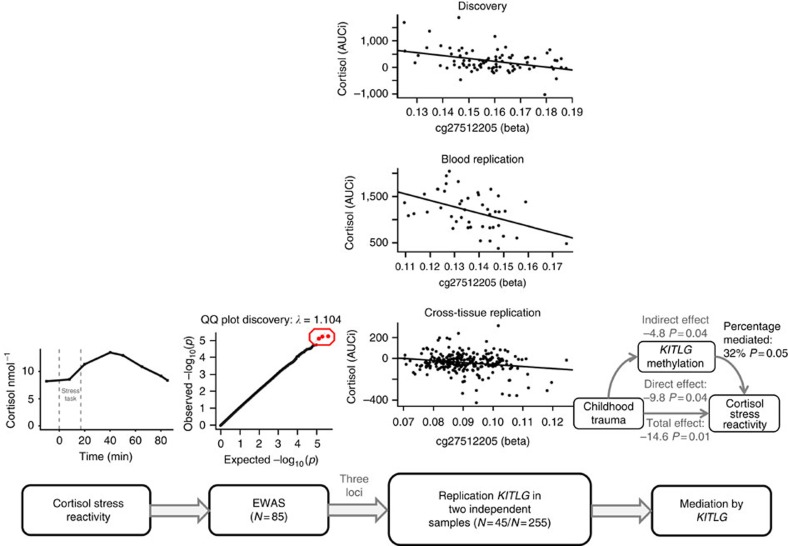
Flowchart of the main analysis. First we performed a genome-wide analysis of the association between cortisol stress reactivity and DNA methylation in the discovery sample (*N*=85). On the basis of the *P* value distribution, we sought replication of the top three loci in two independent samples (*N*=45/*N*=255) and replicated the negative association between the top *KITLG* locus and cortisol stress reactivity. Then we investigated the influence of childhood trauma on *KITLG* methylation and cortisol stress reactivity in the discovery and blood replication sample. On finding an association for childhood trauma with *KITLG* methylation and cortisol stress reactivity in the discovery sample and Caucasian of the blood replication sample, we examined whether the *KITLG* locus is a mediator for the blunted cortisol stress response after childhood trauma exposure.

**Figure 2:**
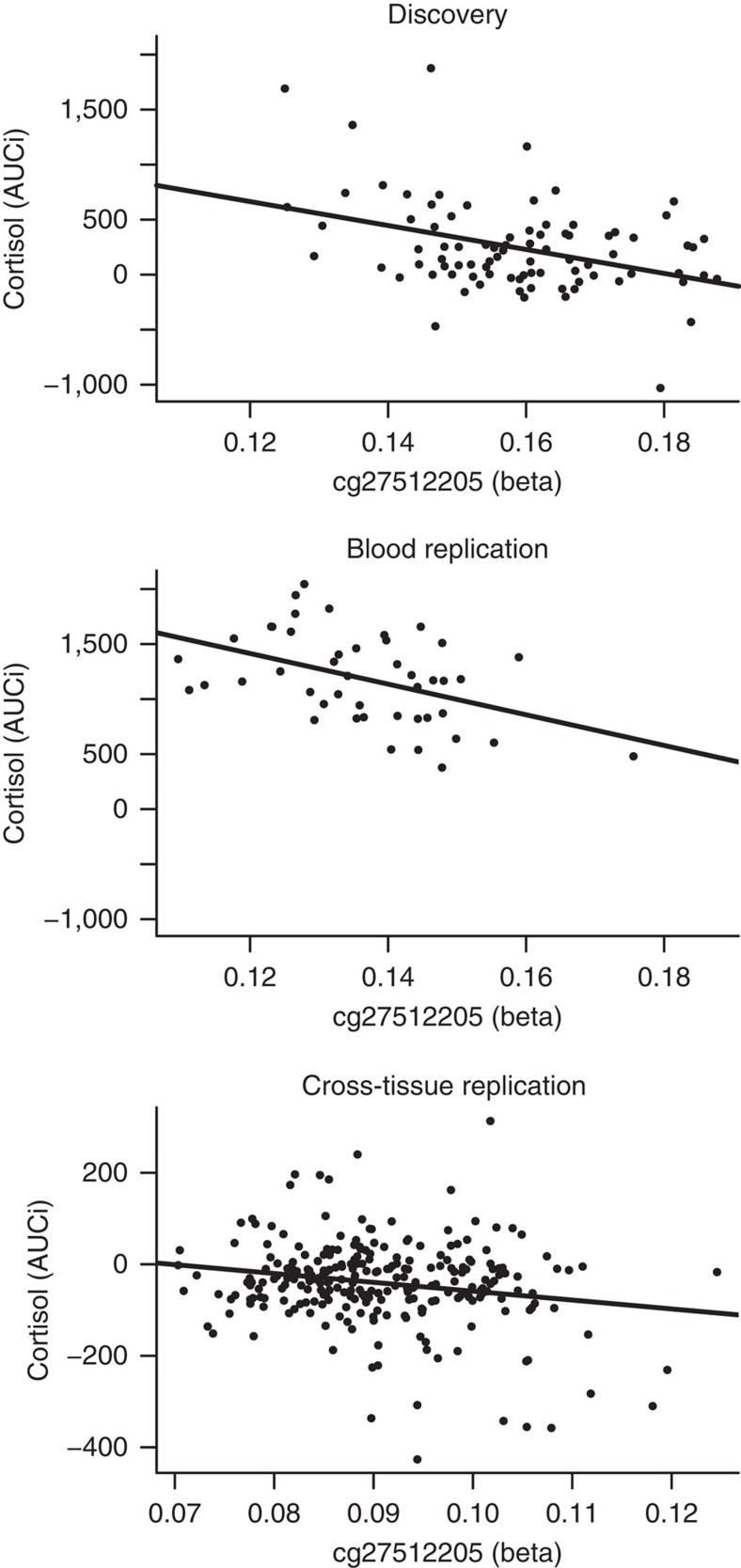
The association between cortisol stress reactivity and DNA methylation at the KITLG/cg27512205 locus in the discovery (top panel), blood replication (middle panel) and cross-tissue replication (bottom panel) samples. A linear regression line is plotted through the individual methylation values.

Our workflow is listed in Fig. 1. After quality control, 385,882 DNA methylation loci were investigated for their association with cortisol stress reactivity (Supplementary Data 1 shows the results for the 22,425 loci with *P* values <0.05 in a linear regression model). Since none of the CpG sites survived adjustment for multiple testing, we selected the three loci that stood out in the *P*-value distribution of the genome-wide cortisol stress reactivity analysis (for QQ plot see Supplementary Fig. 1; cg27512205 *B*=−1,162, *P*=5.8 × 10^−6^; cg05608730 *B*=−935, *P*=6.0 × 10^−6^; cg26179948 *B*=−1,009, *P*=8.0 × 10^−6^ in linear regression models) and were associated with childhood trauma (*P*<0.05 in linear regression models; Supplementary Table 2). The Kit ligand (*KITLG*) locus showed the strongest association with cortisol stress reactivity (cg27512205 chr12: 88579621; *B*=−1,162, *P*=5.8 × 10^−6^, empirical *P* value=2 × 10^−6^, model fit: F(3,81)=15.28, *P*=5.8 × 10^−8^, *R*^2^=0.34 in a linear regression model). This locus was also negatively associated with cortisol stress reactivity in two independent replication samples: a blood replication sample (*N*=45; *B*=−1,039, *P*=0.005, model fit: F(5,39)=5.6, *P*=0.0005, *R*^2^=0.35 in a linear regression model) and a cross-tissue replication sample that used mouth swaps to obtain buccal DNA (*N*=255; *B*=−104, *P*=0.004, model fit: F(3,251)=3.5, *P*=0.02, *R*^2^=0.03 in a linear regression model; Table 1; Fig. 2). Visual inspection of cortisol stress reactivity measures pointed to five potential outliers in the discovery sample. However, Cook's distance was lower than 1 in all analyses, suggesting that these observations did not affect the results (Supplementary Fig. 2).

Moreover, removal of these five potential outliers did not affect the association of cortisol stress reactivity with either childhood trauma (before removal: *B*=−14.6, *P*=0.007; after removal: *B*=−9.0, *P*=0.01 in a linear regression model) or *KITLG* methylation (before removal: *B*=−1,161, *P*=5.8 × 10^−6^; after removal: *B*=−617, *P*=7.0 × 10^−4^ in a linear regression model). To quantify the chance of finding these *P* values in the three independent samples, we used Fisher's method to calculate the combined *P* value of the three samples, yielding an overall significance level of *P*=5.9 × 10^−8^ for the association between cortisol stress reactivity and methylation at the *KITLG* locus.

### Ethnicity and cortisol stress reactivity

**Figure 3:**
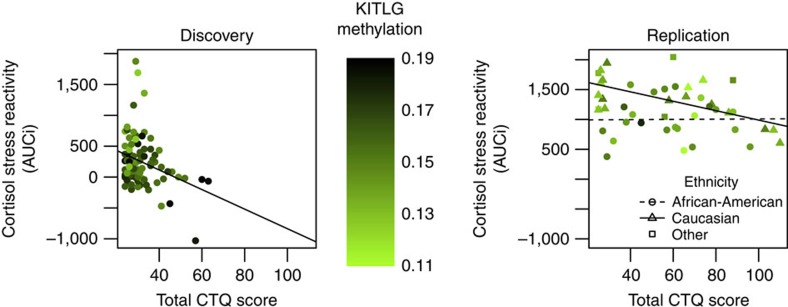
Correlation between childhood trauma (total CTQ score) and cortisol stress reactivity (AUCi) in the discovery and replication sample. Colour indicates methylation levels at the cg27512205 (*KITLG*) locus. In the replication sample (right panel) differences in ethnicity are visualized. AUCi, area under the curve (AUC) with respect to the increase; CTQ, Childhood Trauma Questionnaire.

In light of the influence of current major depressive disorder (MDD)^18^ and ethnicity on cortisol stress reactivity^19^,^20^, we examined the contribution of these factors to our results in the blood replication sample, which included non-Caucasian individuals (Table 2). In the blood replication sample, cortisol stress reactivity was significantly lower in the African-American than the Caucasian individuals (*B*=−300, *P*=0.02, model fit: F(4,40)=4.1, *P*=0.007, *R*^2^=0.22 in a linear regression model; Supplementary Fig. 3), though not related to current MDD (*B*=−19, *P*=0.89 in a linear regression model). Therefore, we performed stratified analyses for ethnicity (Fig. 3; Supplementary Note 1 and Supplementary Fig. 4). In Caucasian individuals (*N*=17), there was a significant negative association between cortisol stress reactivity and methylation of the *KITLG* locus (*B*=−1,961, *P*=0.02, model fit: F(3,13)=2.8, *R*^2^=0.26 in a linear regression model; Supplementary Fig. 4). Moreover, childhood trauma was significantly associated with blunted cortisol stress reactivity only in the Caucasian individuals (*B*=−7.9, *P*=0.003, model fit: F(3,13)=4.8, *P*=0.02, *R*^2^=0.42 in a linear regression model; Fig. 3) and increased methylation at the *KITLG* locus (*B*=0.002, *P*=0.04, model fit: F(8,8)=2.3, *R*^2^=0.39 in a linear regression model). Inclusion of all ethnicities (*N*=45) rendered the relation between childhood trauma and cortisol stress reactivity nonsignificant (*B*=−3.7, *P*=0.09 in a linear regression model), as well as the association of childhood trauma with *KITLG* methylation (*B*=0.001, *P*=0.17 in a linear regression model).

On the basis of these findings in the blood replication sample, we examined the influence of ethnicity in the cross-tissue replication sample with a sensitivity analysis. The association between cortisol stress reactivity and methylation at the cg27512205 locus did not change after inclusion of non-Caucasian individuals and addition of ethnicity as a covariate (*N*=267; *B*=−101, *P*=0.004, F(4,262)=2.5, *P*=0.04, *R*^2^=0.02 in a linear regression model).

### Mediation by *KITLG* methylation

**Figure 4:**
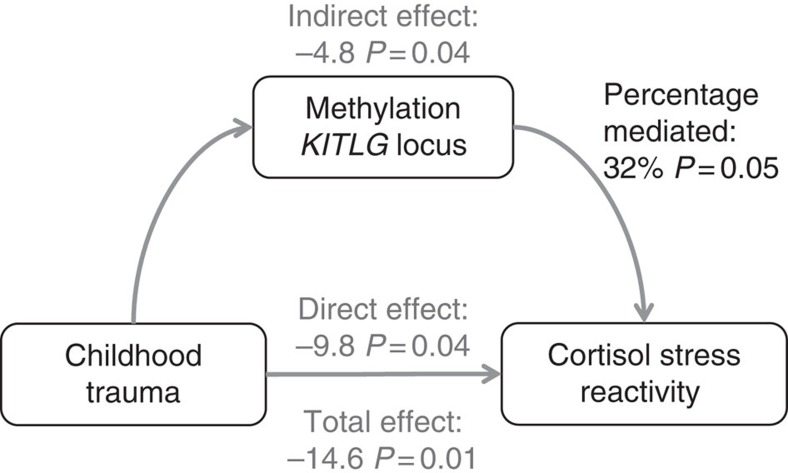
Model used to investigate mediation by the *KITLG* locus in the discovery sample. For graphical representation only, we did not add the sex and age covariates that were included in all statistical analyses.

We carried out an in-depth analysis of the potentially mediating role of *KITLG* methylation in association between childhood trauma and cortisol stress reactivity (Fig. 4). In the discovery sample childhood trauma was associated with lower cortisol stress reactivity (childhood trauma *B*=−14.7, *P*=0.007, model fit: F(3,81)=8.9, *P*=3.8 × 10^−5^, *R*^2^=0.22 in a linear regression model; Fig. 3) and increased DNA methylation at the *KITLG* locus (childhood trauma *B*=0.0045, *P*=0.04, model fit: F(3,81)=1.8, *P*=0.15, *R*^2^=0.03 in a linear regression model). Moreover, the *KITLG* cg27512205 locus mediated 32% of the influence of childhood trauma on cortisol stress reactivity in the discovery sample (indirect effect=−4.8, *P*=0.04; total effect=−14.6, *P*=0.01; proportion mediated=0.32, *P*=0.05 in the mediation model; Fig. 4).

Although in the blood replication sample childhood trauma was significantly associated with *KITLG* methylation and cortisol stress reactivity in the Caucasian individuals (*N*=17), the *KITLG* locus did not mediate the relationship between childhood trauma and cortisol stress reactivity (indirect effect=−1.8, *P*=0.31; total effect=−7.9, *P*<0.001; proportion mediated=0.22, *P*=0.31 in the mediation model). Moreover, mediation by the *KITLG* locus could not be established in the complete replication sample (*N*=45; indirect effect=−1.1, *P*=0.20; total effect=−3.7, *P*=0.09; proportion mediated=0.26, *P*=0.24 in the mediation model).

### Influence of the age of trauma exposure

In the blood replication sample, we found no evidence that age of onset of trauma affected the association between *KITLG* methylation, childhood trauma and cortisol stress reactivity. The age of first trauma exposure (both general and specific trauma) did not alter the association between childhood trauma and cortisol stress reactivity (age of general trauma × childhood trauma interaction *B*=1.9, *P*=0.16 in a linear regression model; age of specific trauma × childhood trauma interaction *B*=1.3, *P*=0.18 in a linear regression model). Similarly, the association between *KITLG* DNA methylation and childhood trauma was not influenced by the age of childhood trauma (age of general trauma × childhood trauma interaction *B*=−0.0006, *P*=0.26 in a linear regression model; age of specific trauma × childhood trauma interaction *B*=−0.0006, *P*=0.14 in a linear regression model). In support, adding age of trauma as a covariate interacting with childhood trauma did not improve linear regression model fits (measured as a reduction of residual sum of squares) for either cortisol stress reactivity as outcome (age of general trauma *P*=0.18; age of specific trauma *P*=0.12) or *KITLG* DNA methylation as outcome (age of general trauma *P*=0.38; age of specific trauma *P*=0.53). In the discovery sample, adult trauma (Life Stressor Checklist-Revised (LSC-R) score mean=3.4, s.d.=2.1) was not significantly associated with cortisol stress reactivity (*B*=−23, *P*=0.28 in a linear regression model) or DNA methylation of the *KITLG* locus (*B*=0.005, *P*=0.60 in a linear regression model), indicating that adult trauma has less impact on cortisol stress reactivity and *KITLG* methylation levels than childhood trauma.

### Blood–brain correlation of the *KITLG* locus

Blood cg27512205 methylation levels were positively correlated to cg27512205 methylation levels in the prefrontal cortex (PFC; *r*=0.293, *P*=0.01 in a linear regression model) and negatively to cg27512205 methylation levels in the superior temporal gyrus (STG; *r*=−0.267, *P*=0.02 in a linear regression model; Supplementary Fig. 5). No significant correlations were found for the entorhinal cortex and the cerebellum (*P*<0.05 in a linear regression model; Supplementary Fig. 5).

### Histone mark analysis of the *KITLG* locus

**Figure 5:**
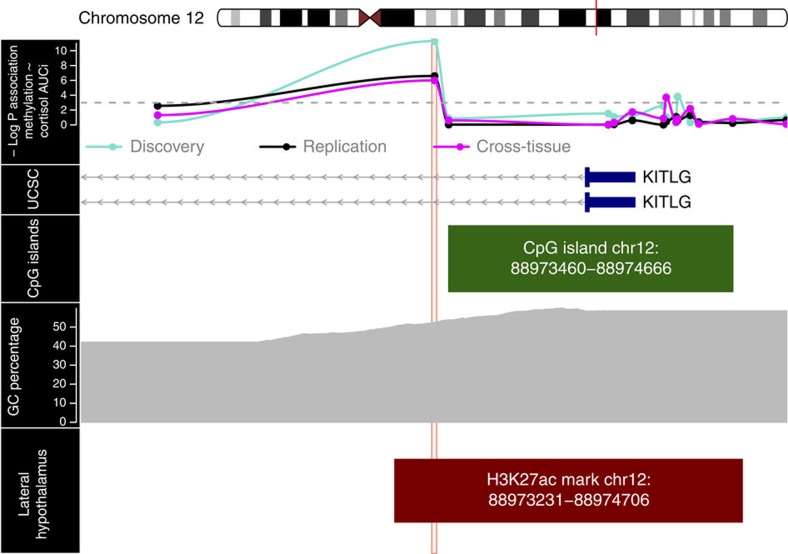
Overview of the 1,500 base pair area downstream and upstream of the cg27512205 *KITLG* locus. The top panel contains the −log *P* values for the association between DNA methylation and cortisol stress reactivity in the discovery (blue, *N*=85), blood replication (black, *N*=45) and cross-tissue replication (magenta, *N*=255) samples per locus (total of 14 loci in the depicted area). The other panels indicate the presence of coding exons (blue blocks) and non-coding introns (grey line) of the *KITLG* gene (second panel), location of a CpG island (third panel) and the percentage of G (guanine) and C (cytosine) bases (fourth panel) in the area around the cg27512205 locus extracted from the UCSC website^70^ with the Gviz R package^62^. The bottom panel indicates the location of a H3K27ac histone modification in lateral hypothalamus tissue (data from ref. 22). Our locus of interest is shaded across all panels by a red rectangle. In the top panel all points above the horizontal dashed grey line are nominally associated (*P*<0.05) loci in a linear regression model. chr, chromosome.

To further analyse the potential functional relevance of the DNA containing the cg27512205 probe, we compared its location to the location of epigenomic signatures that typically cover functional non-coding DNA^21^. We identified enrichment for histone 3 lysine 27 acetylation (H3K27ac), overlapping the cg27512205 probe location in lateral hypothalamus tissue (Fig. 5). This histone mark was previously found to be selectively present at active gene regulatory DNA suggesting that our probe is located in a functional sequence^22^.

### Regional analysis of *KITLG* gene methylation

Cg27512205 was the only *KITLG* probe (out of 18 present on the methylation array) associated with cortisol stress reactivity in all the three independent samples (Fig. 5).

### *KITLG*-related methylation network analyses

**Figure 6:**
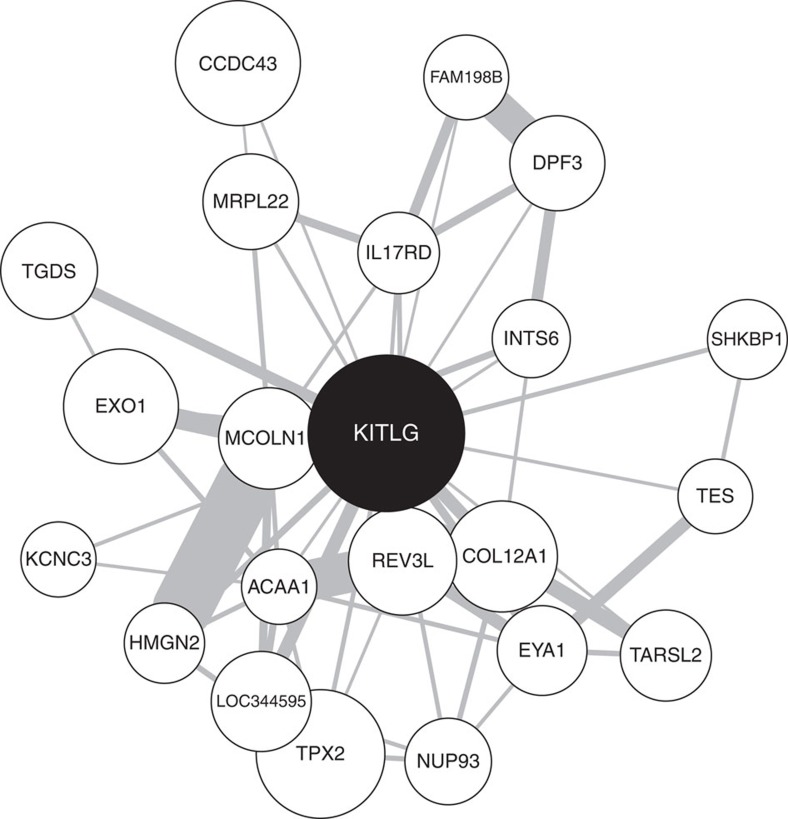
Graphical depiction of the connection between the *KITLG*-related probe and its direct neighbours within the red module (Supplementary Data 2). The node size indicates the association with cortisol stress reactivity, while the width of the lines indicates the connection strength between the nodes.

By using weighted gene co-expression network analysis (WGCNA), we derived 40 consensus modules, based on the correlation patterns among probes in the discovery and cross-tissue samples. These 40 consensus modules were significantly related to cortisol stress reactivity in the discovery sample (multiple analysis of covariance (MANCOVA) Pillai's trace=0.72, F(40,37)=2.4, *P*=0.004) and borderline significant in the cross-tissue replication sample (MANCOVA Pillai's trace=0.21, F(40,212)=1.4, *P*=0.051). Subsequently, we analysed the module containing the *KITLG* probe (the ‘red' module; Fig. 6 and Supplementary Data 2). This red module was significantly associated with cortisol stress reactivity in both the discovery (F(1,76)=4.4, *P*=0.04 in the follow-up analysis of variance (ANOVA)) and the cross-tissue replication sample (F(1,251)=4.3, *P*=0.04 in the follow-up ANOVA).

To further understand the biology of the red module, we examined enrichment for gene ontology (GO) terms and the potential regulation by microRNAs (miRNAs) of the *KITLG* network within the red module. The red module contained 21,211 probes linked to 9,494 genes, which were significantly enriched for GO terms related to metabolism and regulation of transcription (Supplementary Data 2). Two thousand seven hundred forty-eight of these probes were nominally associated with cortisol stress reactivity in the discovery sample. Selection of the 5% strongest connections yielded a 21-gene network around the *KITLG* probe (Fig. 6). With the webGestalt tool, we found that the 21-gene network around *KITLG* is a preferred target for three miRNAs: miR449 (genes *COL12A1*, *SHKBP1* and *KITLG* FDR_hypergeometric_ (FDR, false discovery rate)=0.0012), miR23A/miR23B (genes *EYA1*, *HMGN2* and *KITLG* FDR_hypergeometric_=0.0018) and miR9 (genes *COL12A1*, *CCDC43* and *KITLG* FDR_hypergeometric_=0.0019; Supplementary Table 3). The entire red module (containing 9,494 of 19,815 genes) was enriched for genes related to these three miRNAs (miR449 Fisher's exact test, odds ratio (OR)=1.4, *P*=0.0015, FDR=0.009, miR23A/miR23B Fisher's exact test, OR=1.4, *P*=7.3 × 10^−6^, FDR=9.8 × 10^−5^ and miR9 Fisher's exact test, OR=1.3, *P*=9.5 × 10^−5^, FDR=0.0006). Several other methylation modules were also enriched for these miRNAs (10 modules enriched for miR449 Fisher's exact test FDR<0.05; 19 modules enriched for miR23A/miR23B Fisher's exact test FDR<0.05 and 20 modules enriched for miR9 Fisher's exact test FDR<0.05; Supplementary Table 4–6).

## Discussion

By using a unique and unbiased approach, we analysed the relationship between DNA methylation levels and cortisol stress response in three independent samples (total *N*=385) using an experimental stress paradigm. Genome-wide analysis of the association of whole blood DNA methylation with cortisol stress reactivity in the discovery cohort identified a locus (cg27512205) in the *KITLG* gene. This locus was also associated with cortisol stress reactivity in two independent samples: one replication sample in blood and another replication sample using buccal cell DNA. Even though the observed DNA methylation differences in *KITLG* were small, the impact was considerable since the model accounted for 35% of the variation in cortisol stress reactivity in the discovery and the blood replication sample. Moreover, *KITLG* methylation was a mediator in the association between childhood trauma and cortisol stress reactivity. The identified methylation locus (cg27512205, chr12: 88579621) is located on the north shore of a CpG island near the *KITLG* gene (Fig. 5). This gene codes for a ligand of the C-kit receptor and is involved in cellular developmental processes such as hematopoiesis by activating the C-kit receptor^23^. The involvement of KITLG protein in stress-induced HPA-axis activity is biologically plausible, because KITLG levels correlate with glucocorticoid receptor expression in response to *in vitro* stress-induced erythropoiesis^24^. In mice, early life stress increased both anxiety and KITLG expression in the hippocampus^25^. Also, C-kit-positive hematopoietic progenitors proliferate in response to chronic stress, resulting in higher levels of inflammatory leukocytes in mice^26^.

Recent studies highlight the complexity of epigenetic regulation and indicate that the interplay between DNA methylation and enhancers may trigger cascades of transcriptional events that are highly relevant for neurodevelopment^21^,^27^,^28^. The identified *KITLG* locus is located in a region enriched for the histone mark H3K27ac in the human hypothalamus, which is pivotal for cortisol stress reactivity^22^; this supports a biologically relevant and functional signal. H3K27ac is typically found at active regulatory DNA, such as enhancer and promoter regions, and is associated with a more open chromatin structure indicative of gene transcription^21^,^22^,^27^,^28^. Interestingly, the cg27512205 CpG is the only *KITLG* probe located both in the H3K27ac-enriched region and on the shore of a CpG island. As CpG island shores are frequently linked to DNA methylation differences that affect gene transcription and expression^29^, the co-occurrence of these two epigenetic signatures suggests that methylation differences at this CpG location could alter gene regulatory DNA near *KITLG*. Interestingly, young animals exposed to early life stress altered histone modifications at the *KITLG* promoter, specifically an increase in H3K9ac and a decrease of the repressive H3K9me; this change is associated with increased hippocampal KITLG expression^25^. Another potential insight into the biological mechanisms related to *KITLG* methylation comes from our co-expression network analyses showing that *KITLG* is part of a gene network enriched for genes regulated by miRNAs 449 (miR449), 23A/23B (miR23A/miR23B) and 9 (miR9). Notably, in rodents two of these miRNAs were previously linked to stress system functionality^30^,^31^. Specifically, miR449 is involved in the regulation of corticotropin-releasing factor type 1 receptor in the anterior pituitary and HPA-axis activation^31^. Also, miR9 is upregulated in the frontal cortex of mice in response to acute stress^30^.

The fact that the *KITLG* locus was significantly related to cortisol stress reactivity in three independent samples is noteworthy considering the substantial differences in study characteristics between these samples. The blood replication sample was smaller, ethnically diverse and included individuals selected for either low or high levels of childhood trauma. In addition, cortisol was measured in blood and, even though differences in cortisol stress reactivity can be detected in both blood and saliva^3^, this may have contributed to the difference in cortisol values between the discovery and blood replication sample. The cross-tissue replication sample measured methylation in DNA extracted from buccal cells in relatively young participants (15–18 years) and used a public speaking task without an arithmetic stressor. Despite these differences, the *KITLG* locus was in all cases related to cortisol stress reactivity, which supports the robustness of the observation. This is further supported by the fact that a significant association between cortisol stress reactivity and *KITLG* methylation was observed in buccal and blood DNA. Some recommend buccal samples for population epigenetic studies, as they contain more hypomethylated DNA regions, which tend to cluster around disease associated single-nucleotide polymorphisms (SNPs)^32^; others, however, argue that demographic factors may be better reflected in blood DNA methylation patterns^33^. Blood and buccal cells are peripheral tissues and do not necessarily reflect changes in DNA methylation in the central nervous system. However, there are several reasons why *KITLG* methylation in peripheral tissues can be informative for the neurobiological mechanisms underlying cortisol stress reactivity. First, cortisol is released into the periphery by the pituitary and is known to affect multiple tissue types. Second, DNA methylation co-expression network analyses with the module containing the *KITLG* probe (red module) demonstrated that this module was associated with cortisol stress reactivity in both tissue types, suggesting that a broader methylation network around *KITLG* is biologically relevant for stress reactivity. Previous reports on a variety of traits such as age^34^ also indicate that methylation co-expression networks are stable indicators for epigenetic regulation across tissue types. Third, HPA-axis genes are abundantly expressed in peripheral blood mononuclear cells^35^. Peripheral changes in methylation may therefore at least partially be a proxy of epigenetic processes in the brain. Indeed, previous studies have shown that childhood trauma-related changes in methylation obtained from peripheral blood mononuclear cells were significantly enriched for central nervous system pathways^16^. From the four brain areas that we examined, significant correlations with blood methylation were found in the PFC and the STG, which are biologically relevant brain regions for stress. Thus, a wealth of literature points to the PFC as a pivotal regulator of the stress response (for review see ref. 36); in agreement, altered cortisol stress responses have been found after lesions in the PFC^37^. Regarding the STG: a recent meta-analysis supported a link of this area to stress susceptibility^38^. In light of the opposing blood–brain correlations, it may be hypothesized that the STG and PFC have opposing roles in the regulation of cortisol stress reactivity, but this cannot be inferred from the present study and warrants further research.

It is particularly interesting that epigenetic regulation of the stress response was found to be related to childhood trauma. In the discovery sample, increased levels of childhood trauma were significantly related to blunted cortisol stress reactivity and higher methylation at the *KITLG* locus (Fig. 4). In the blood replication sample, a similar result was only obtained in Caucasian individuals. In the complete blood replication sample, this association was (just) not significant, suggesting that ethnic diversity influences analyses on the relationship between childhood trauma and cortisol stress reactivity. This may be the result of overall lower cortisol stress reactivity in Afro-American individuals^19^,^20^. Unlike the discovery sample, there was no evidence that *KITLG* methylation is a mediator of the association between childhood trauma and cortisol stress reactivity in either the entire (*N*=45) or Caucasian-only (*N*=17) replication sample. Overall non-replication of the mediation analysis may be due to a more heterogeneous ethnicity, smaller sample size—below the recommended *N*=50 (ref. 39)—and unfavourable distribution of childhood trauma due to the inclusion of individuals based on either low or high levels of childhood trauma (Supplementary Fig. 6).

The relationship between childhood trauma and a blunted cortisol response in the present study is in concordance with some but not all of the published literature (16 studies; Supplementary Table 1). For example, in the discovery sample and in Caucasian individuals from the replication sample, the explained variance of cortisol stress reactivity by childhood trauma was 34 and 26%, respectively. This is comparable to the 30% explained variance in the study of Carpenter *et al*.^5^ who also included healthy individuals without a psychiatric diagnosis. Previous studies have pointed to specific periods during which individuals are particularly sensitive to trauma exposure^40^. However, in the blood replication sample, we did not find any indications that our *KITLG* results were related to age of onset of childhood trauma. Moreover, in the discovery sample, cortisol stress reactivity and *KITLG* methylation were not significantly related to traumatic experiences in adult life, suggesting that early life is a more sensitive period for the persistent impact of trauma on HPA-axis activity.

In conclusion, this study shows that altered stress reactivity following childhood trauma in humans is related to altered DNA methylation levels at the *KITLG* locus. Identification of such epigenetic marks may help to identify inter-individual differences in susceptibility to traumatic stress in early life and elucidate the neurobiological pathways underlying its long-lasting detrimental effects.

## Methods

### Study population

For discovery, 85 healthy individuals were recruited from the general population at the University Medical Center, Utrecht, The Netherlands (see Table 1 for sample characteristics). Participants had three or more Dutch grandparents, were not taking any prescription medication and had not been enroled in stress-related research before participation. The absence of any mental or physical disorder was confirmed by an independent rater in an interview according to the Mini-International Neuropsychiatric Interview (MINI) plus criteria^41^. Participants abstained from heavy meals, drinks other than water or heavy exercise for at least 2 h before the study protocol. Current use of psychoactive substances (amphetamines, 3,4-methylenedioxy-methamphetamine (MDMA), barbiturates, cannabinoids, benzodiazepines, cocaine and opiates) was assessed by self-report and verified with a urine multi-drug screening device (InstantView).

The blood replication sample consisted of 45 individuals who were part of a larger study, the Conte Center Study for the Psychobiology of Early-Life Trauma (MH58922) and included some individuals with exposure to childhood trauma before the age of 13 years and with/without a diagnosis of MDD^42^ (Table 1). Depressed mood was assessed with the 21-item self-report Beck Depression Inventory (BDI)^43^. Eleven subjects with a score above 9 on the BDI were classified as current MDD. Exclusion criteria include: current medical illness, lifetime diagnosis of psychosis or bipolar disorder, alcohol or substance abuse within 6 months or eating disorders within the last year. None of the participants was receiving psychiatric treatment or currently taking psychiatric medication. Heavy smokers (>20 cigarettes per day) were excluded. The blood replication sample was ethnically more diverse and included predominantly Afro-American (*N*=23, 52%) and Caucasian (*N*=17, 38%) individuals.

The cross-tissue validation sample consisted of 255 healthy adolescents participating in the longitudinal RADAR-Y (Research on Adolescent Development and Relationships Young cohort) study (Table 1). DNA samples were collected for 414 subjects of whom 314 completed the stress task. Exclusion criteria were adolescents who currently received prescription medication (*N*=42) or were of non-Caucasian ethnicity (*N*=17).

All studies were approved by an ethical review board and performed according to the ICH guidelines for Good Clinical Practice and the Declaration of Helsinki. More specifically, the discovery and RADAR-Y studies were approved by the medical ethical committee of the University Medical Centre Utrecht, while the Conte Center Study for the Psychobiology of Early-Life Trauma was approved by the Institutional Review Board of Emory University School of Medicine. For all studies, participants gave written informed consent before inclusion and were financially compensated. The data used to replicate the findings of the discovery sample are available in Supplementary Data 3.

### Stress procedure

Both the discovery and blood replication sample used a version of the Trier Social Stress Test (TSST) as a stress induction task, consisting of a public speaking test (PST) and subsequent arithmetic task. In the discovery study, the TSST was adapted to a group format and carried out as previously described^44^. Cortisol levels were measured with an in-house radioimmunoassay in eight saliva samples (Salivette) collected over a time period of 90 min (Supplementary Fig. 7)^44^. In the blood replication study, an individual TSST was conducted^45^. Cortisol levels were examined in blood samples obtained from an indwelling venous catheter during eight 15-min intervals (15 min before the TSST up to 90 min afterwards). Blood was collected into chilled EDTA-coated Monovette and centrifuged immediately before cortisol was measured with a radioimmunoassay. In the cross-tissue sample, a PST based on the Leiden-PST was used^46^. Cortisol levels over a time period of 45 min were measured with a radioimmunoassay in seven saliva samples obtained by passive drooling into a plastic tube (0.5 ml SaliCap)^46^.

In all studies, participants were tested in the afternoon to mitigate the influence of diurnal variations in cortisol secretion and the area under the curve (AUC) with respect to the increase (AUC_i_) of cortisol was calculated based on the consecutive data points as previously described^47^.

### Trauma exposure

In the cross-tissue sample, childhood trauma exposure was not measured. In the discovery and blood replication sample, childhood trauma exposure was assessed using the short version of the Childhood Trauma Questionnaire (CTQ)^48^. The validity of the 25 clinical CTQ items, including a Dutch translation, has been demonstrated in clinical and population samples^48^,^49^. In the discovery sample, one translated item (‘I believe I was molested') was excluded as this translation was found to be an invalid indicator of childhood sexual abuse in a previous validation study^49^. In the blood replication sample, age of onset of general trauma and age of onset of any specific childhood trauma exposure were assessed with the Early Trauma Inventory^50^. In the discovery sample, data on adult trauma (>16 years) were available for 69 of 85 individuals who completed the LSC-R self-report questionnaire^51^.

### DNA methylation measurement

In all studies genome-wide DNA methylation levels were assessed using Illumina Infinium HumanMethylation450K BeadChip (Illumina) arrays. X chromosome, Y chromosome and nonspecific binding probes were removed^52^. Failed probes were excluded based on a detection *P* value >0.001 and bead count <5. In addition, probes with SNPs of minor allele frequency >5% within 10 base pairs of the primer were excluded after constructing ancestry estimates based on their principal components as proposed by Barfield *et al*.^53^ (list of CpG sites is available at http://genetics.emory.edu/research/conneely/annotation-r-code.html). In the discovery sample 385,882 DNA methylation probes survived quality control and were used for further genome-wide analysis. Finally, in all studies DNA methylation data were normalized and batch effects were removed based on inspection of the association of the principal components of the methylation levels with plate, Sentrix array and position using multivariate linear regression and visual inspection of heat maps (see Supplementary Figs 8–10 and Supplementary Notes 1–3 for the model summaries per sample). Quality control and analysis were performed with the wateRmelon^54^, the Minfi^55^, the Limma^56^ and the sva packages^57^ from the Bioconductor platform in R.

More specifically, in the discovery sample whole blood was obtained before the stress test and DNA was extracted with the Gentra Puregene kit (Qiagen, Valencia, CA, USA). DNA concentration and integrity was assessed using Ribogreen and Bioanalyzer. Bisulphite conversion was conducted with Zimo kits (Zymo Research, Orange, CA, USA) using standard procedures. Samples were distributed over the twelve 450K arrays according to gender and age to reduce batch effects to the minimum. Intensity read outs, quality control parameters and methylation measures were obtained from the GenomeStudio software. In total, 20,845 probes with failed detection in more than 1% of the participants or <5 beads in 5% of samples were excluded. All samples were included as none of the samples had more than 1% of probes failed^54^. The data were normalized to remove systematic differences in overall signal distribution due to probe design bias using the Beta MIxture Quantile dilation (BMIQ) normalization^58^ as implemented in the wateRmelon package^54^. After removing batch effects of Sentrix array and position with the Combat procedure from the sva package no batches were apparent^59^ (Supplementary Fig. 8). Finally, cell-type composition estimates (another well-known potential confounder in whole blood samples) were calculated using a Minfi-based implementation of the Houseman algorithm^55^ with FACS-sorted DNA methylation data as a reference set and related to DNA methylation levels (see Supplementary Fig. 8 and Supplementary Note 2 for model summary).

In the replication study, blood was obtained before the stress test. DNA was extracted and genome-wide DNA methylation levels were assessed using Illumina 450K DNA methylation arrays as previously published^14^,^16^. Intensity read outs, normalization, cell-type composition estimation, beta and *M* values were obtained using the Minfi package (version 1.10.2) in Bioconductor^55^. In total, 233 failed probes were excluded based on a detection *P* value >0.01 in at least 75% of the samples. We removed probes located within 10 bp from a SNP with a minor allele frequency of ≥0.05 in any of the populations represented in the sample. The data were then normalized with functional normalization; an extension of the quantile normalization procedure implemented in the Minfi R package^55^. Sentrix array and position-related batch effects were identified by linear regression analysis with the first principal component of the methylation levels and visual inspection of principal component analysis (PCA) plots. Batch effect removal was performed with the Combat procedure as implemented in the sva package^59^.

In the cross-tissue study, buccal swaps were obtained before the PST and DNA was extracted using the chemagic saliva isolation kit on a Chemagen Module I workstation (Chemagen Biopolymer Technologie AG, Baesweiler, Germany). Samples were equally distributed over fifteen 450K arrays according to gender and age to reduce batch effects to the minimum. Intensity read outs, quality control parameters and methylation measures were obtained using the methylumi package (version 2.14.0) in Bioconductor^60^. In total, 3,574 probes with failed detection in more than 1% of the participants or <5 beads in 5% of samples were excluded as were three samples where more than 1% of probes failed^54^. The three excluded samples were already identified before data analysis, as there were technical difficulties during bisulphite conversion. Therefore, technical duplicates were present on the array and we obtained high-quality methylation data for all participants in the cross-tissue study. The data were normalized to remove systematic differences in overall signal distribution due to probe design bias using BMIQ^58^ as implemented in the wateRmelon package^54^. After removing batch effects related to Sentrix array and position with the Combat procedure from the sva package no remaining batches were apparent^59^ (see Supplementary Note 3 for model summaries; Supplementary Figs 9 and 10).

### Blood–brain sample

For the identified *KITLG* methylation locus, we compared blood–brain correlation in a database containing 78 individuals (40–105 years old) described in more detail in a previous study^61^. In short, whole blood samples were collected before death, as well as PFC (*N*=74), entorhinal cortex (*N*=69), STG (*N*=75) and/or cerebellum (*N*=69) tissue post mortem. Approximately, 500 ng DNA from each sample was extracted and assessed using 450K Illumina DNA methylation arrays. Raw signals were extracted with Illumina GenomeStudio software and further pre-processed with the methylumi and wateRmelon^54^ packages in R. Initial quality control checks were performed using functions in the methylumi package to assess concordance between reported and genotyped gender. Non-CpG SNP probes on the array were also used to confirm that all four brain regions and matched bloods were sourced from the same individual. Array data for each of the tissues was normalized separately using the dasen function from the wateRmelon^54^ package and initial analyses were performed separately by tissue. The effects of age and sex were regressed out before blood and brain methylation levels were compared using linear regression modelling as previously described^61^.

### Histone mark in hypothalamus

H3K27ac data determined with ChIP-sequencing analysis on post-mortem hypothalamus tissue was downloaded from a previous study^22^ and overlaid with our probe data. Enrichment was found at the identified cg27512205 probe in the *KITLG* locus and visualized using the Gviz R package^62^.

### Statistical analyses

All statistical analyses were carried out in R version 3.2.2 (ref. 63). All regression modelling was performed with the Limma^64^ package and outliers were defined using Cook's distance with a cutoff value of 1. We report the regression coefficient (*B*) and *P* value for all analyses. If relevant individual parameters have a significant association (*P*<0.05), we also report the percentage of variance explained by the complete model (*R*^2^) with the corresponding F statistic and *P* value. Beta values of methylation were used for graphical display, but analyses were conducted with *M* values (log_2_ ratio of methylation probe intensity) for better statistical validity^65^. To account for potential confounding by blood cell type in the discovery and whole blood replication sample (Supplementary Note 1 and Supplementary Note 2), we calculated standardized residuals for the *M* values using cell count estimates from the Houseman algorithm as the independent variables. Because cortisol stress reactivity and methylation levels may vary with age and sex^19^,^66^, both factors were included as covariates in all analyses. In previous studies, both current MDD^18^ and ethnicity^19^,^20^ influenced cortisol stress reactivity. In light of the ethnic diversity and current MDD individuals in the blood replication sample, we investigated whether cortisol stress reactivity was associated with current MDD or ethnicity. If there was a significant association with either current MDD or ethnicity, stratified analyses were performed and the determinant (current MDD or ethnicity) was included as covariate in all analyses of the complete blood replication sample. In the Caucasian discovery sample population, stratification did not play a role, therefore the methylation-based population principal components as proposed in the Barfield study^53^ were not included as covariate (Supplementary Fig. 8). In the cross-tissue replication sample, non-Caucasian individuals (*N*=12) were excluded a priori to make the cross-tissue replication sample more comparable to the discovery sample regarding ethnicity. Moreover, we also performed a sensitivity analysis by including the 12 non-Caucasian individuals (*N*=267) and adding ethnicity as a covariate.

To investigate DNA methylation and cortisol stress reactivity, we first performed a genome-wide association analysis in the discovery sample with cortisol stress reactivity (AUCi) as the outcome and DNA methylation, age and sex as the determinants in a linear regression model. We considered a FDR at the 0.05 level as genome-wide significant. Visual inspection of the QQ plot in the discovery sample did not indicate a deviant distribution of *P* values (*λ*=1.10; Supplementary Fig. 1). On the basis of the *P* value distribution, we sought replication for loci of which the strength of the association stood out. In the two independent replication samples, we implemented the same linear regression model with cortisol stress reactivity as dependent and methylation, age and sex as indicators. On the basis of the association with cortisol stress reactivity (Supplementary Note 1), ethnicity was added as covariate to the model in the whole blood replication sample. Finally, we interrogated potential type-I error inflation for the replicated methylation loci in the discovery sample by calculating an empirical *P* value based on 1,000,000 label-swapping permutations.

To examine the influence of age at trauma exposure in the discovery sample, adult trauma was also associated with either cortisol stress reactivity or DNA methylation of the identified locus using linear regression models with sex and age as covariates. In the replication sample, we also investigated whether age of onset of childhood trauma modified its relationship with either cortisol stress reactivity or DNA methylation of the identified locus. To this end, the interaction between age of trauma with childhood trauma levels was examined in linear regression models with DNA methylation or cortisol stress reactivity as outcomes and sex, age and ethnicity as covariates.

In all samples, we investigated the association between cortisol stress reactivity and DNA methylation of the other probes located on the *KITLG* gene using a linear regression model with age and sex as covariates. On the basis of the association with cortisol stress reactivity (Supplementary Note 1), ethnicity was added as covariate to the model in the whole blood replication sample.

We hypothesized that DNA methylation of the *KITLG* locus mediates the association between childhood trauma and cortisol stress reactivity. Therefore, we investigated the association between childhood trauma and cortisol stress reactivity using a linear regression model with sex and age as covariates. Next, the association between childhood trauma and methylation was investigated. On the basis of the association with cortisol stress reactivity (Supplementary Note 1), ethnicity was added as covariate to the model in the whole blood replication sample.

To quantify the average causal mediation effect of DNA methylation, we performed a model-based mediation analysis in the discovery and replication sample, using the mediation package in R^67^. This method uses the information of two linear regression models: (1) DNA methylation as outcome and childhood trauma, age and sex as determinants and (2) cortisol stress responsivity as the outcome variable and DNA methylation, childhood trauma, age and sex as determinants. The algorithm estimates the presence of mediation (average causal mediation effect/indirect effect) as well as the proportion of the link between childhood trauma and cortisol stress reactivity mediated by DNA methylation by using a quasi-Bayesian Monte Carlo method with 10,000 simulations.

Finally, in the discovery and cross-tissue samples, weighted gene co-expression network analysis was performed with the WGCNA package in R to identify consensus methylation clusters^34^. We did not use the blood replication sample for the identification of consensus methylation clusters, since the sample size was relatively small with a heterogeneous background with regard to ethnicity and current depression. The consensus clusters containing loci of interest were further characterized based on their relationship with cortisol stress reactivity and biological processes. To link the methylation cluster to biological processes a GO-term-enrichment analysis was conducted with the missMethyl^68^ package in R. First, all loci surviving quality control were mapped to genes. Next, the relationship between the number of probes per gene and the probability of selection was calculated with an adapted GOseq method by Young *et al*.^69^ Finally, the probes in the module of interest were selected and the other loci used as a reference set to perform a modified version of a hypergeometric test to incorporate the over-representation of the selected genes in each GO category.

To examine the association between the WGCNA methylation modules and cortisol stress reactivity in the discovery and cross-tissue replication samples, we performed MANCOVA with the participant score on the methylation consensus modules as outcomes and cortisol stress reactivity, sex and age as determinants. For the discovery sample, cell count estimates were also added as covariates. Separate follow-up ANOVA analyses were carried out for the individual modules containing loci of interest, but only if the methylation modules were significantly associated to cortisol stress reactivity.

To establish the connections of the replicated loci within these methylation clusters, we selected all probes with a nominal association to cortisol stress reactivity in the module containing a replicated locus. Then connection strength was established based on the correlation between individual loci and only the top 5% strongest connections were used for plotting and enrichment analysis. On the basis of these criteria, the neighbours of the replicated locus were selected and evaluated for miRNA regulation using the WebGestalt tool based on the miRTarBase website (http://mirtarbase.mbc.nctu.edu.tw/). Enrichment for genes related to a particular miRNA was investigated with a Fisher's exact test for the presence of the selected genes present in the module of interest compared to the presence of the selected genes present in all other modules.

## Additional information

**Accession codes:** The data of the discovery study have been deposited in NCBI's Gene Expression Omnibus under the accession number GSE77445.

**How to cite this article:** Houtepen, L. C. *et al*. Genome-wide DNA methylation levels and altered cortisol stress reactivity following childhood trauma in humans. *Nat. Commun.* 7:10967 doi: 10.1038/ncomms10967 (2016).

## Supplementary Material

Supplementary Information Supplementary Figures 1-10, Supplementary Tables 1-6, Supplementary Notes 1-3 and Supplementary References

Supplementary Data 1 Table containing a list of all the probes nominally associated with cortisol stress reactivity (AUCi) in the discovery sample with the regression coefficient and p-value.

Supplementary Data 2 List of all the Gene Ontology (GO)-terms significantly enriched (FDR<0.05) in the KITLG-probe containing red Weighted Gene Coexpression Network Analysis (WGCNA) methylation cluster.

Supplementary Data 3 Excel file with three sheets, one per dataset (discovery, blood-replication and buccal-replication). Each sheet contains phenotype data and, for the top 3 loci (cg27512205, cg05608730 and cg26179948), the DNA methylation values used for analyses (after normalization and batch effect correction) as well as signal intensities and detection p-values. Detection p-values represent the confidence that a given transcript is expressed above the background defined by negative control probes. Abbreviations: Cort_AUCi= cortisol stress response area under the curve (AUC) with respect to the increase, CTQ= Childhood Trauma Questionnaire, LSCR= Life Stressor Checklist- Revised, CD8T= CD8 T cell proportion, CD4T=CD4 T cell proportion, NK=Natural Killer cell proportion, Mono=Monocytes cell proportion, Gran=Granulocytes cell proportion, Bcell= B cell proportion. The mention of ‘beta' after the cg number (e.g. cg27512205_beta), stands for ‘DNA methylation levels expressed in beta'. Similarly, ‘meth', ‘unmeth' and ‘det_pval' indicate respectively the methylated, unmethylated and detection p-value for the specified probe (e.g. cg27512205_unmeth).

## Figures and Tables

**Table 1:** Characteristics of the replicated kit ligand (KITLG) locus from the cortisol stress reactivity epigenome-wide association study (EWAS).

Cg number	27512205
Gene	*KITLG*
Location	Chr 12: 88579621 north-shore CpG island
Discovery mean methylation* (range)	0.15 (0.12–0.19)
Replication mean methylation* (range)	0.14 (0.11–0.18)
Cross-tissue mean methylation* (range)	0.09 (0.07–0.12)
Discovery association cortisol AUCi	*B*=−1,161, *P*=5.8 × 10^−6^†
Blood replication association cortisol AUCi	*B*=−1,040, *P*=0.006†
Cross-tissue association cortisol AUCi	*B*=−104, *P*=0.003†
Discovery association CTQ	*B*=0.005, *P*=0.04†
Blood replication association CTQ	*B*=0.001, *P*=0.146

AUCi, area under the curve (AUC) with respect to the increase; CTQ, Childhood Trauma Questionnaire.

^*^Methylation in percentage (beta).

^†^Denotes a nominal association in a linear regression model (*P*<0.05).

**Table 2:** Sample description.

Characteristic	Discovery sample (*N*=85)	Blood replication sample (*N*=45)	Cross-validation sample (*N*=255)
Sex (% of female)	50.5	80	45
Age (mean in years, range)	33 (18 to 69)	28 (19 to 45)	17 (15 to 18)
Caucasian ethnicity (%)	100	38	100
Current MDD (%)	0	24	NA
Childhood trauma (mean total score, range)	31.9 (24 to 63)	56.8 (25 to 110)	NA
Cortisol stress reactivity (mean AUCi, range)	242.3 (−1,030 to 1,876)	1,185 (378 to 2,045)	−37 (−426 to 313)

AUCi, area under the curve (AUC) with respect to the increase; MDD, major depressive disorder; NA, not applicable.
