# Supplementary material for: Genome-wide DNA methylation levels and altered cortisol stress reactivity following childhood trauma in humans
Source: Nat Commun. 2016 Mar 21;7:10967. doi: 10.1038/ncomms10967 (PMC4802173; doi:10.1038/ncomms10967)
Supplement: Supplementary Information — Supplementary Figures 1-10, Supplementary Tables 1-6, Supplementary Notes 1-3 and Supplementary References [file ncomms10967-s1.pdf]

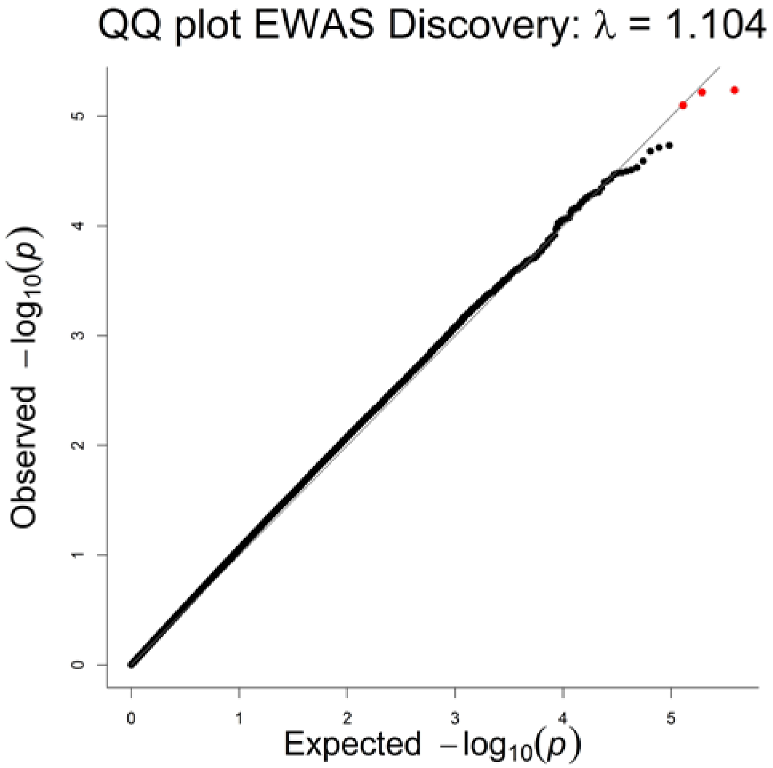

Figure 1 QQplot showing the p-value distribution for the genome-wide cortisol stress reactivity analysis in the discovery sample (N=85). The three methylation loci for which replication was sought are denoted by red dots.

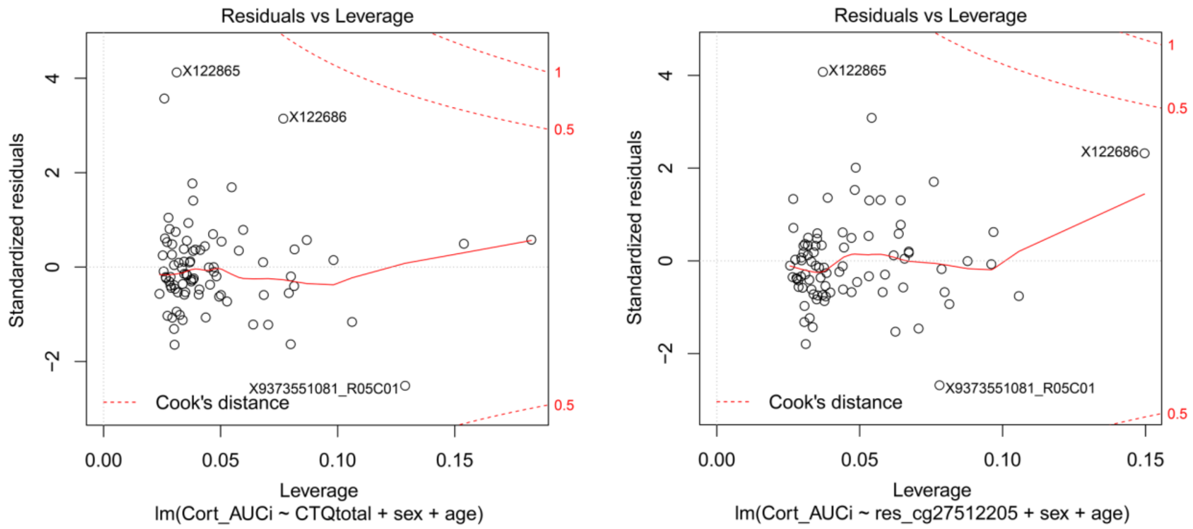

Figure 2 Cook plot of the leverage and standardized residuals for the association between childhood trauma and cortisol stress reactivity (left panel) and for the association between methylation at the KITLG locus and cortisol stress reactivity (right panel) in the discovery sample (N=85).

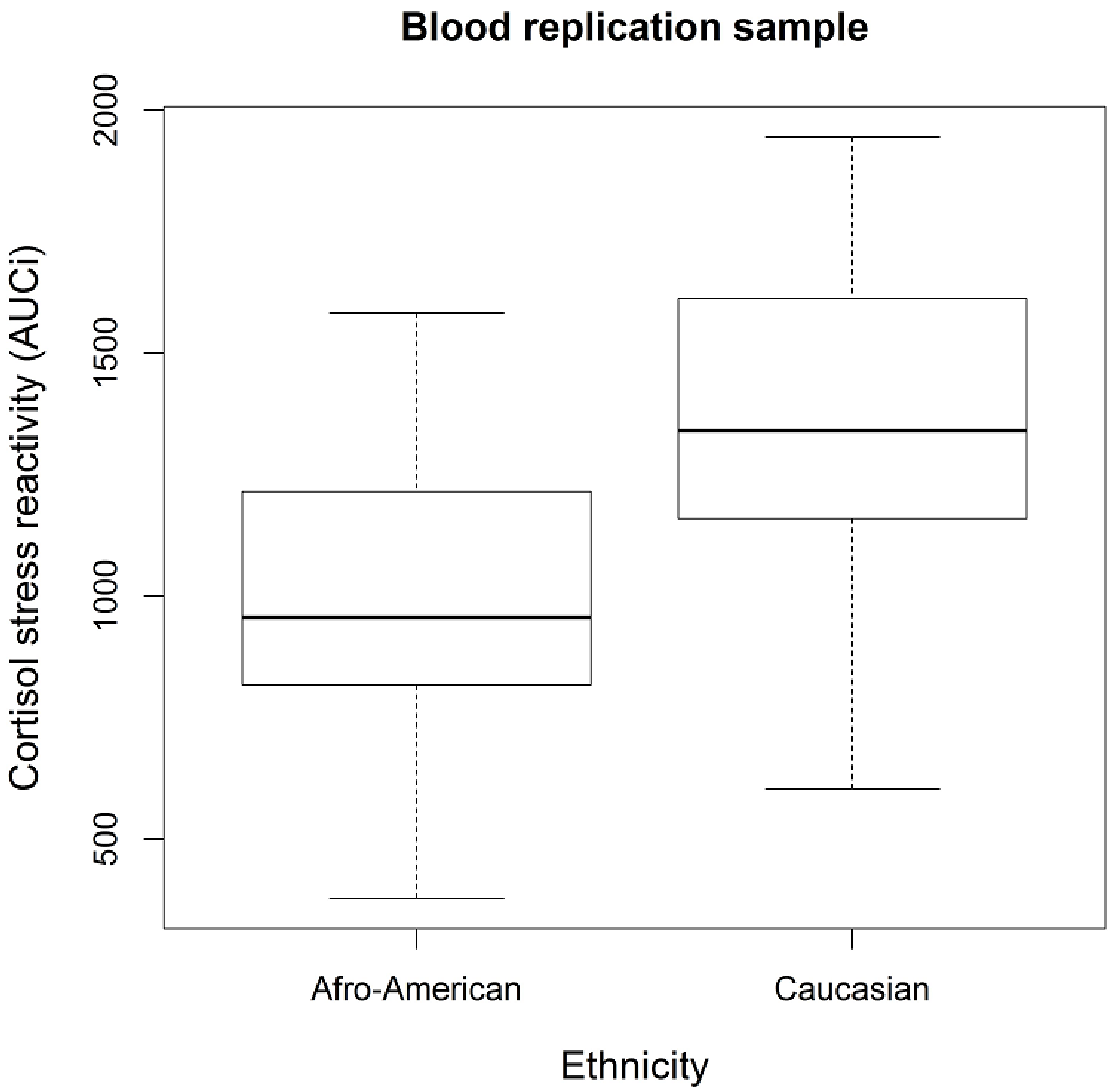

Figure 3 Cortisol stress reactivity (AUCi) is significantly lower in Afro-American individuals (n=23) compared to Caucasian individuals (n=17) (p=0.002 in a linear regression model). In this boxplot the whiskers extend to the most extreme data point which is no more than 1.5 times the length of the box away from the box. Abbreviations: AUCi= Area under the curve(AUC) with respect to the increase.

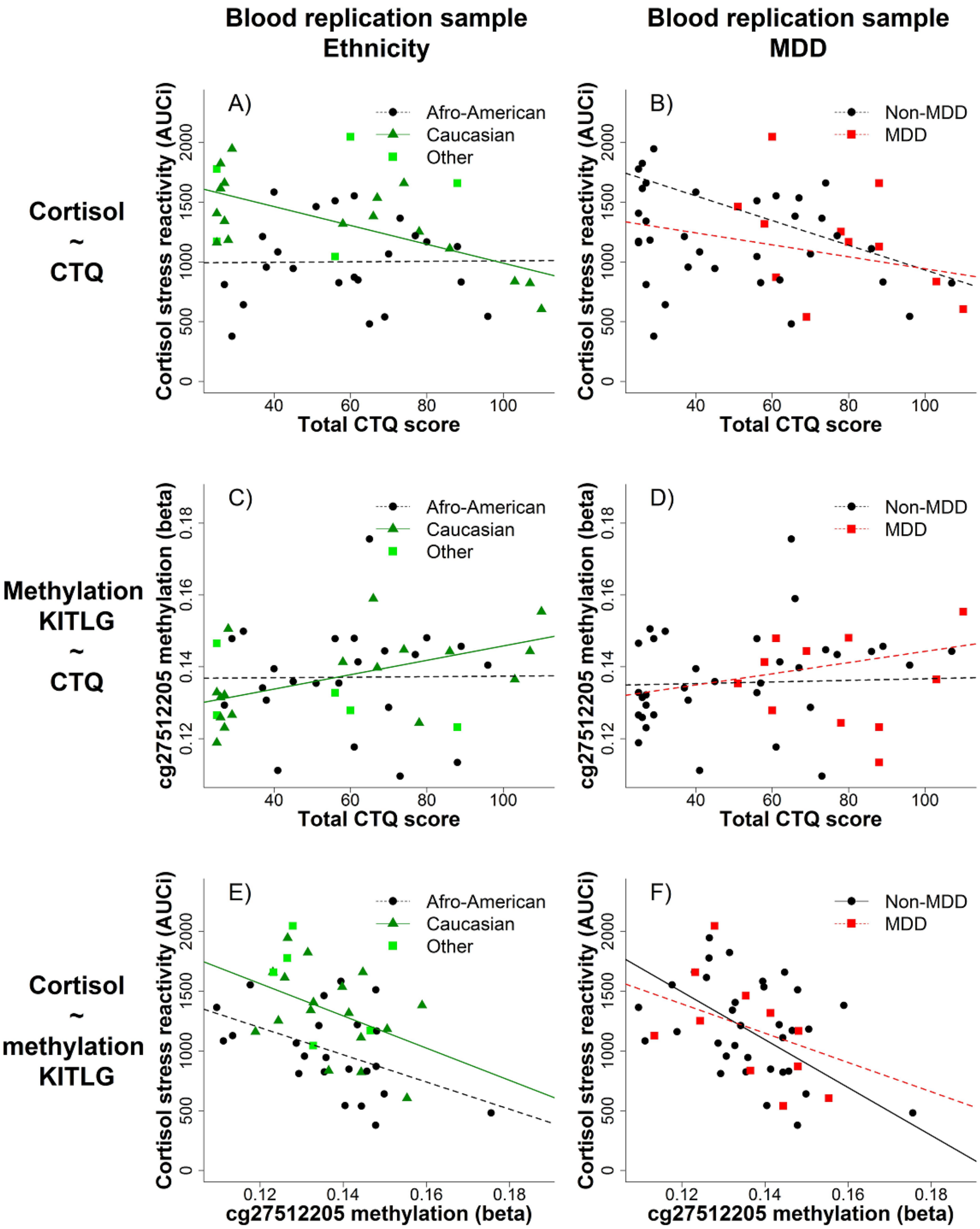

Figure 4 Overview of the stratified analyses for ethnicity and current major depressive disorder (MDD) status in the replication sample (N=45). Significant correlations in linear regression models are present for the Caucasians (N=17; solid green lines in panels A, C, E) and for the non-MDD subjects (N=34; cortisol~methylation only; solid black line in panel F). Dashed lines indicate non-significant correlations. Abbreviations: CTQ= childhood trauma questionnaire, AUCi=Area under the curve (AUC) with respect to the increase.

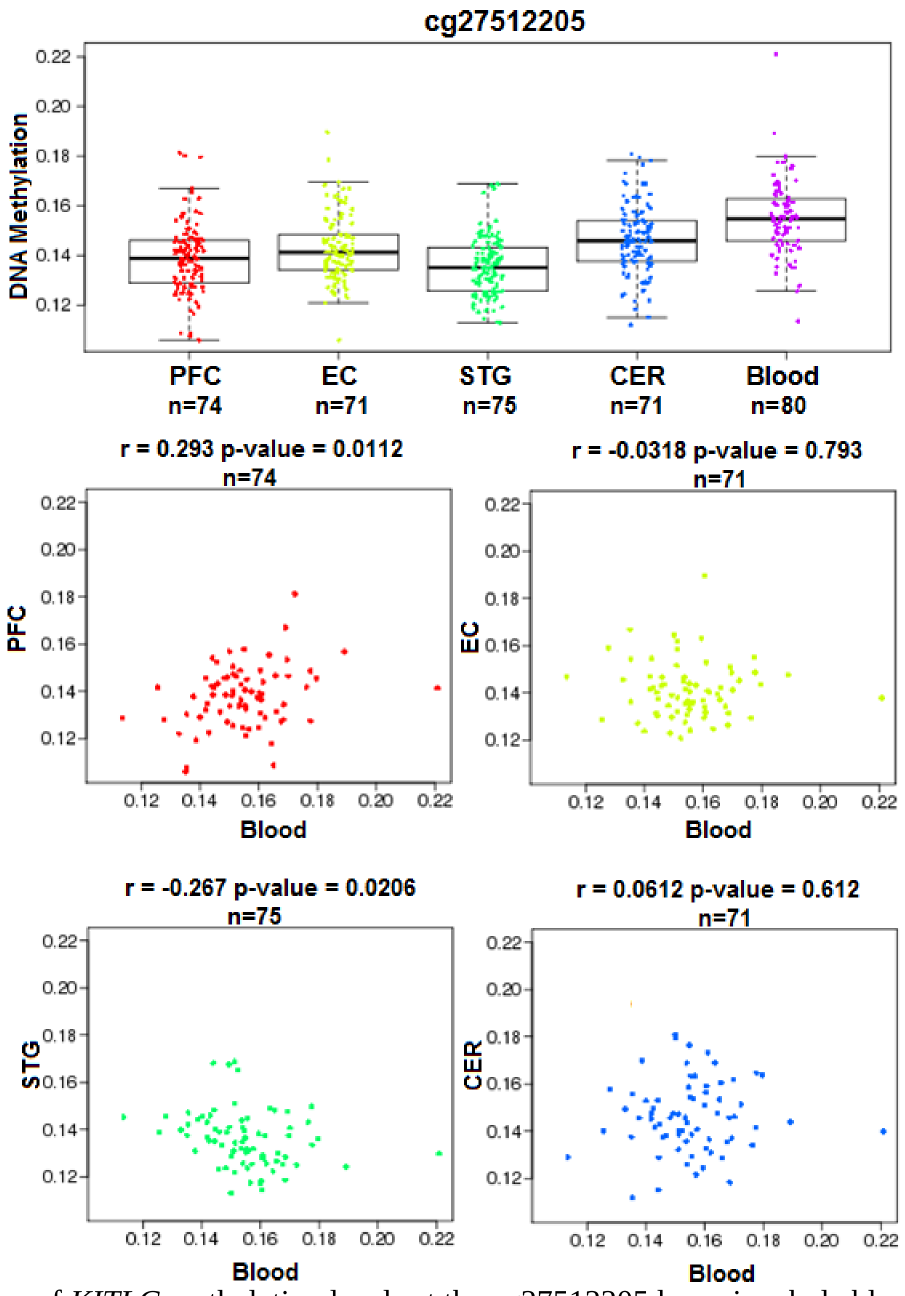

Figure 5 Correlation of *KITLG* methylation levels at the cg27512205 locus in whole blood and four brain regions in a linear regression model. The top panel is a boxplot with the DNA methylation levels per tissue type. In this boxplot the whiskers extend to the most extreme data point which is no more than 1.5 times the length of the box away from the box. The prefrontal cortex (PFC; upper left panel), superior temporal gyrus (STG; lower left panel), entorhinal cortex(EC; upper right panel) and cerebellum (CER; lower right panel).

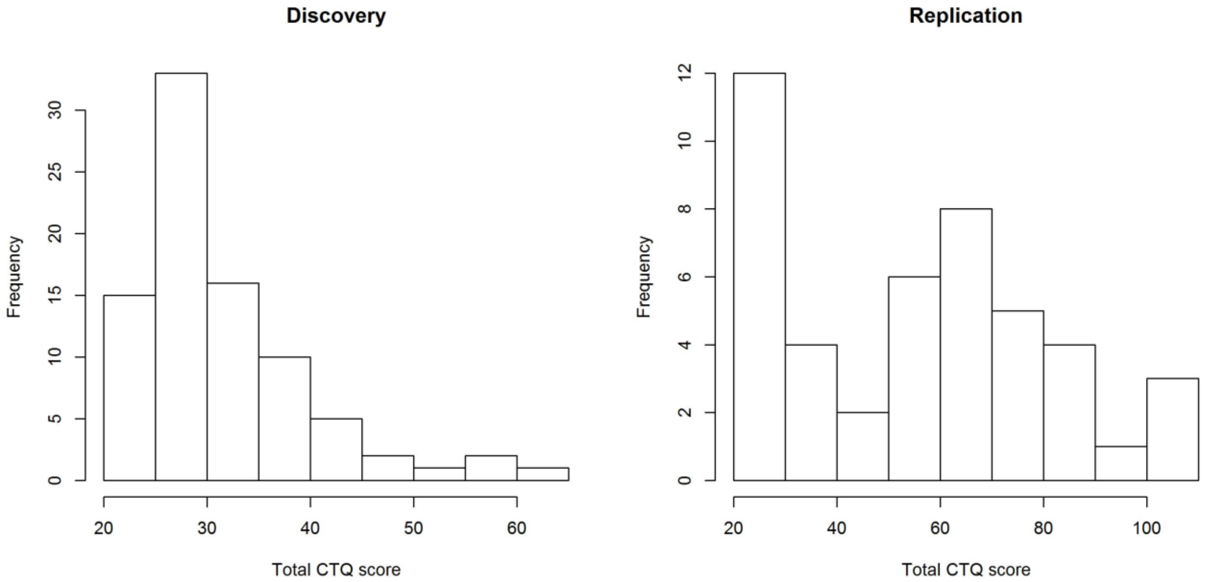

Figure 6 Distribution of the childhood trauma questionnaire (CTQ) scores in the discovery (N=85) and replication (N=45) samples.

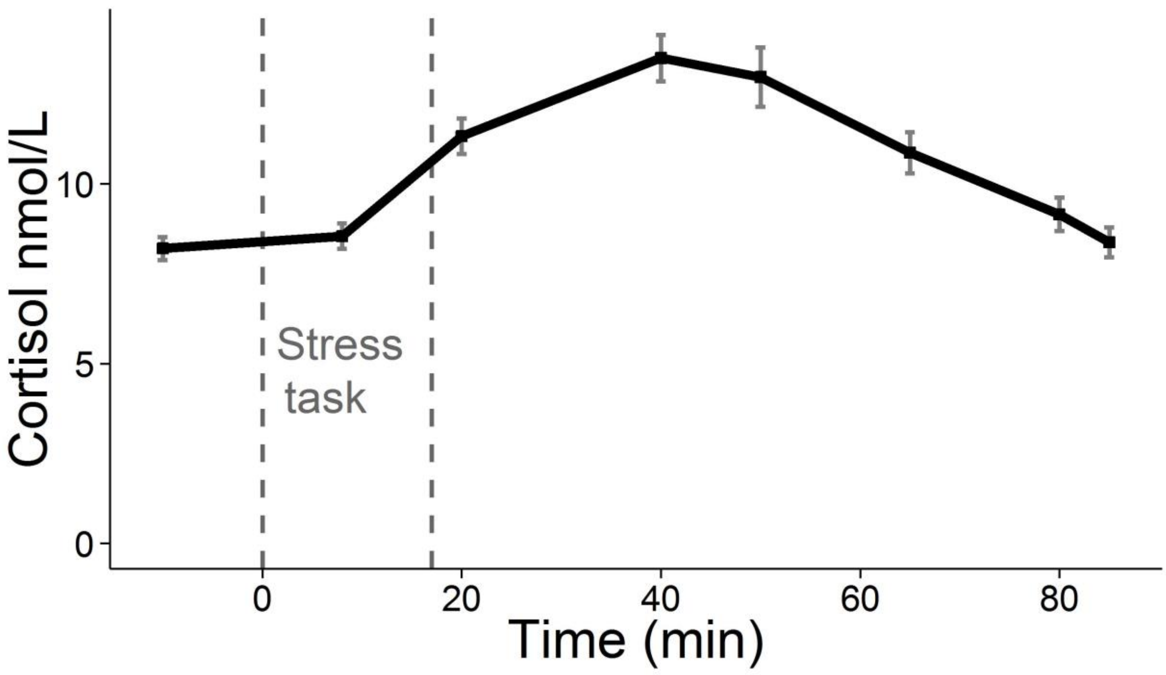

Figure 7 The cortisol response over time in the discovery sample of 85 healthy controls. The error bars indicate the standard error of measurement (SEM) per time point in these 85 healthy controls. Abbreviations: min= minutes.

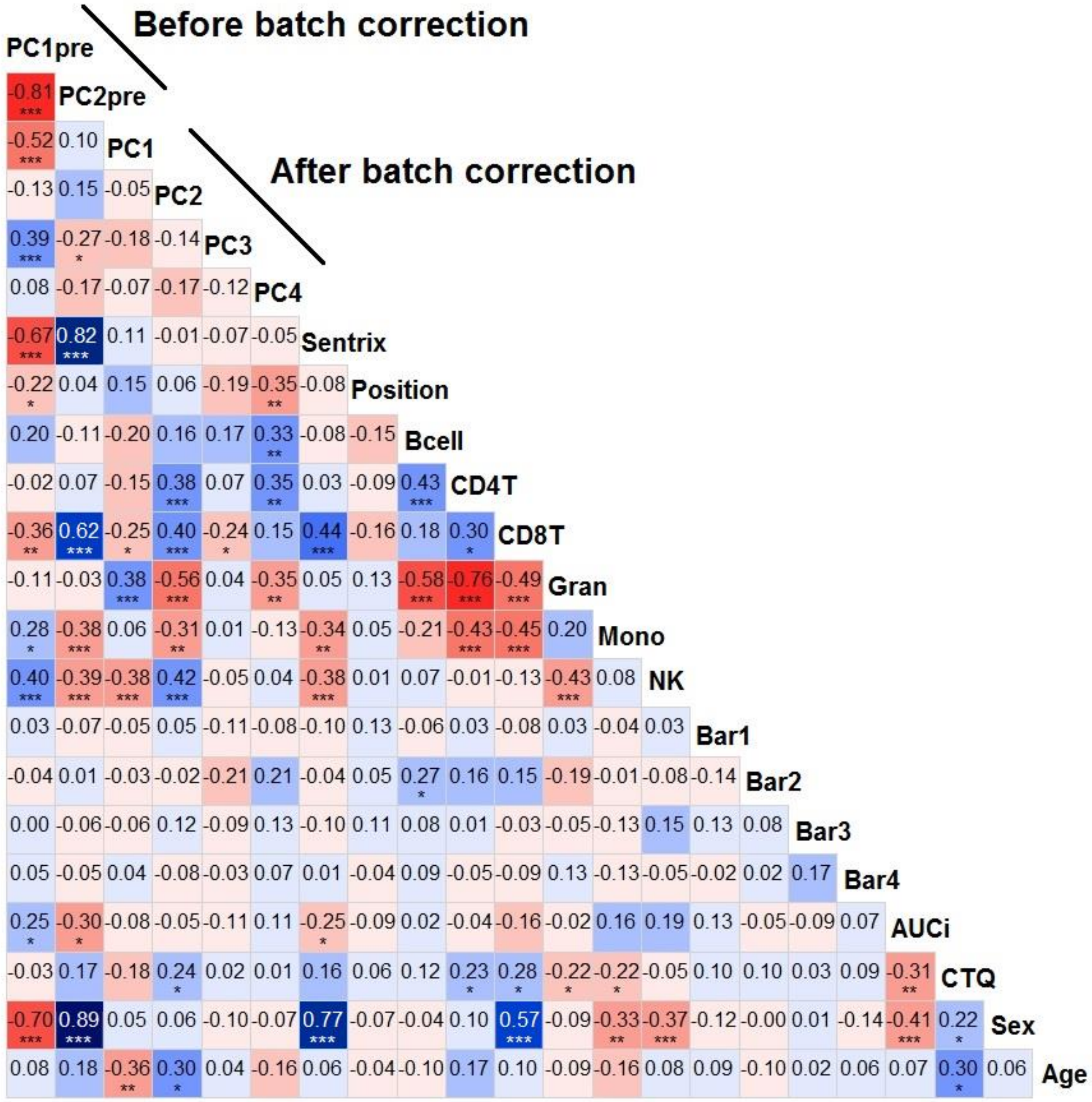

Figure 8 The correlation between batch effects, potential confounders and general methylation levels (principal components) before and after batch effect removal in the discovery sample (N=85). Positive correlations are blue and negative correlations are red, the color intensity is related to the size of the correlation coefficient. Significant values for these Spearman's correlation are denoted by * p<0.05, ** p<0.001, ***p<0.0001. Abbreviations: AUCi= cortisol stress response area under the curve(AUC) with respect to the increase,CTQ= Childhood Trauma Questionnaire, CD8T= CD8 T cell proportion, CD4T=CD4 T cell proportion, NK=Natural Killer cell proportion, Mono=Monocytes cell proportion,Gran=Granulocytes cell proportion, Bar= ancestry estimates calculated according to [1], PC= principal component. The mention of 'pre' after the principal component number (e.g. PC1pre), stands for 'before batch effect removal'.

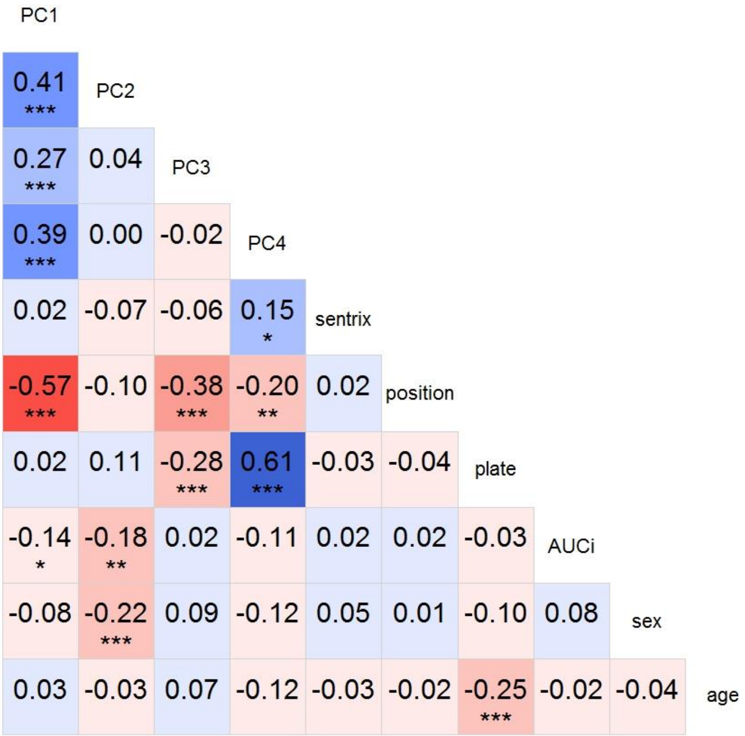

Figure 9 Correlation between our variables of interest and potential confounders before batch correction in the cross-tissue sample (N=255). Significant values for these Spearman's correlation are denoted by * p<0.05, ** p<0.001, ***p<0.0001. Abbreviations: Cort_AUCi= cortisol stress response area under the curve(AUC) with respect to the increase, Col= sentrix column,pc= principal component

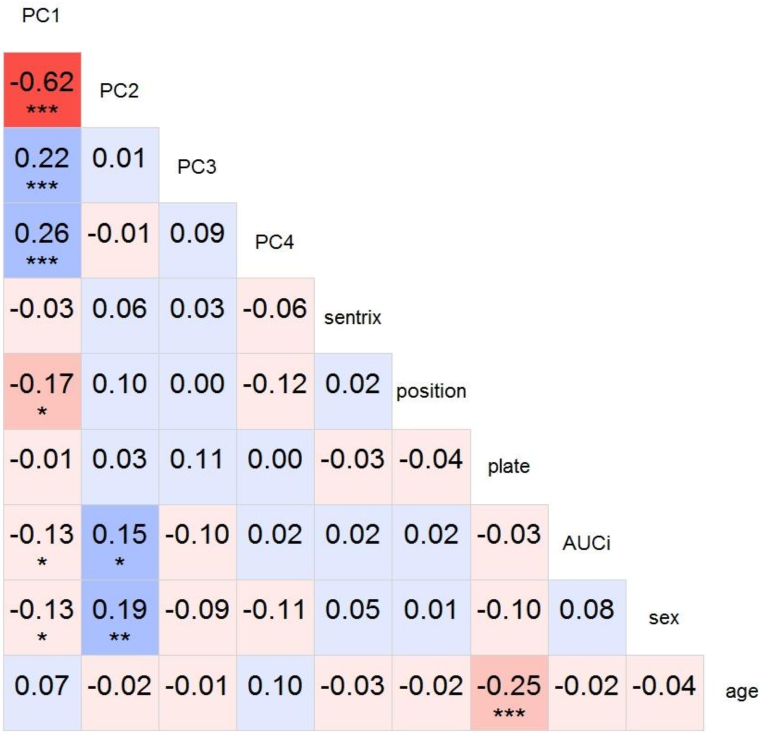

Figure 10 Correlation between our variables of interest and potential confounders after batch correction for sentrix and position in the cross-tissue sample (N=255). Significant values for these Spearman's correlation are denoted by * p<0.05, ** p<0.001, ***p<0.0001. Abbreviations: AUCi= cortisol stress response area under the curve(AUC) with respect to the increase, Col= sentrix column, pc= principal component

Table 1 Overview of studies investigating childhood trauma and cortisol stress reactivity.

Reference	Population (N)	Ethnicity	Stress task	Childhood trauma assessment	Relation childhood trauma and cortisol
(Carpenter et al. 2007)[2]	HC (N=50)	Cauc (76%); Black (6%); Asian (8%); Other (10%)	TSST	CTQ	↓
(Carpenter et al. 2011)[3]	HC (N=78)	Unknown	TSST	CTQ	↓
(Elzinga et al. 2008)[4]	HC (N=80)	Unknown	TSST	TEC	↓
(Heim et al. 2000)[5]	HC (N=26) vs MDD (N=23)	Afr-Am(27%); Cauc (73%)	TSST	ETI	Ns in HC, ↑ in MDD
(Moran-Santa Maria et al. 2010)[6]	HC (N=43) and Cocaine dependence (N=42)	Unknown	TSST	ETI-SR	Ns in HC, Ns in cocaine dependent
(Pesonen et al. 2010)[7]	HC vs WWII separation(N=282)	Unknown	TSST	WWII separation	↑ separated both parents
(Witt et al. 2011)[8]	HC (N=228)	Cauc (99%)	TSST	Enriched family adversity index	N/A focus NPY genotype: ↓ TT allele
(Buchmann et al. 2014)[9]	HC (N=195)	Unknown	TSST	CTQ	N/A focus FKBP5 genotype: ↓ C allele, ns T allele
(Kraft and Luecken ,2009)[10]	HC (N=94)	Cauc(78%); Afr-Am(2%); His(14%); Oth(6%)	Speech	Parental divorce	↓
(Luecken,1998)[11]	HC (N=61)	Cauc(66%); Black(25%); Asian(7%); Other(3%)	Speech/ video	Parental loss	↑
(Otte et al. 2005)[12]	HC (N=76)	Unknown	Video	LSCR interview; age <14 yrs	Ns
(Goldman-Mellor et al. 2012)[13]	HC (N=543)	Unknown	Two 5-min tasks: Stroop test/ Mirror tracing	ELS questions in longitudinal study	↑ELS no distress ↓ELS distress
(Elzinga et al. 2010)[14]	Social Anxiety Disorder(N=50)	Unknown	TSST	TEC	↑SAD patient
(Videlock et al. 2009)[15]	Early life stress mixed sample	Unknown	Sigmoidoscopy	Trauma history	Ns

	(HC& IBS N=83)			questionnaire	
(Bremner et al. 2003)[16]	HC vs PTSD (N=41)	Unknown	20 min Cognitive challenge	ETI	Ns
(Santa Ana et al. 2006)[17]	HC vs PTSD (adult/ childhood index trauma N=89)	Cauc(73%); Afr-Am(25%); Other(2%)	CPT(= Cold pressure test)	CAPS determine index trauma childhood vs adult	Ns

Abbreviations: Cauc=Caucasian, Afr-Am=Afro-American, CTQ= childhood trauma questionnaire, ELS=early life stress, DHS= Daily hassles scale; LES= Life experiences survey; LSCR= Life stressor checklist revised, SAD= social anxiety disorder, MDD= major depressive disorder, HC= healthy control, PTSD= post traumatic stress disorder, IBS= Irritable Bowel Syndrome, TSST= Trier social stress test, ETI= Early trauma inventory; CPT= Cold pressure test; WWII= second world war; Ns= non significant; N/A= not applicable.

Table 2 Characteristics of the top three DNA methylation loci from the genome-wide cortisol stress reactivity methylation analysis.

**Cg number**	**27512205**	**05608730**	**261779948**
Gene	*KITLG*	*C1QTNF2*	*JAZF1*
Location	Chr 12: 88579621<br>North-shore CpG island	Chr 5: 159797775<br>South-shore CpG island	Chr 7: 28181035<br>South-shore CpG island
Discovery (N=85) mean methylation# [range]	0.15 [0.12 - 0.19]	0.38 [0.29 - 0.47]	0.12 [0.09 - 0.16]
Replication (N=45) mean methylation# [range]	0.14 [0.11-0.18]	0.37 [0.31-0.46]	0.10 [0.08-0.14]
Cross-tissue (N=255) mean methylation# [range]	0.09 [0.07-0.12]	0.08 [0.05-0.11]	0.23 [0.16-0.31]
Discovery association cortisol AUCi	B= -1161, p=5.8x10^{-6}*	B= -935, p=6.0x10^{-6}*	B= -1009, p=8.0x10^{-6}*
Blood replication association Cortisol AUCi	B=-1040, p=0.006*	B=433, p= 0.15	B=223, p=0.48
Cross-tissue association cortisol AUCi	B=-104 , p=0.003*	B=38, p=0.13	B=-2, p=0.95
Discovery association CTQ	B=0.005, p=0.04*	B=0.006, p=0.04*	B=0.006, p=0.02*
Blood replication association CTQ	B=0.001, p=0.146	B=-0.0001, p=0.903	B=-1.4 x10^{-5}, p=0.990

* denotes a nominal association (p<0.05) in a linear regression model
# Methylation in percentage (betas)
Abbreviations: AUCi= Area under the curve(AUC) with respect to the increase; CTQ=Childhood trauma questionnaire; *KITLG*=kit ligand; *C1QTNF2*= C1q and tumor necrosis factor related protein 2; *JAZF1*= Juxtaposed with another zinc finger protein 1

Table 3 The webgestalt output for the enrichment analysis of microRNA-related genes in the 19 gene network around *KITLG* (EntrezGene id 4254). We used a hypergeometric test with all Entrez genes as background and a significance level of 0.05 (FDR correction). The results for each enriched gene sets are listed in this table. For each gene set, the first row lists database name, gene set name, and corresponding gene set ID. The second row lists the following statistics: C: the number of reference genes in the category; O: the number of genes in the gene set and also in the category; E: the expected number in the category; R: ratio of enrichment; rawP: p value from hypergeometric test; adjP: p-value adjusted by the multiple test adjustment.

**Database:microRNA Target Name:hsa_CACTGCC,MIR-34A,MIR-34C,MIR-449 ID:DB_ID:673**					
C=277; O=3; E=0.14; R=21.23; rawP=0.0004; adjP=0.0012					
Index	UserID	Gene Symbol	Gene Name	EntrezGene	Ensembl
1	*KITLG*	*KITLG*	KIT ligand	4254	ENSG00000049130
2	*SHKBP1*	*SHKBP1*	SH3KBP1 binding protein 1	92799	ENSG00000160410
3	*COL12A1*	*COL12A1*	collagen, type XII, alpha 1	1303	ENSG00000111799
**Database:microRNA Target Name:hsa_AATGTGA,MIR-23A,MIR-23B ID:DB_ID:683**					
C=417; O=3; E=0.21; R=14.10; rawP=0.0012; adjP=0.0018					
Index	UserID	Gene Symbol	Gene Name	EntrezGene	Ensembl
1	*KITLG*	*KITLG*	KIT ligand	4254	ENSG00000049130
2	*EYA1*	*EYA1*	eyes absent homolog 1 (Drosophila)	2138	ENSG00000104313
3	*HMGN2*	*HMGN2*	high mobility group nucleosomal binding domain 2	3151	ENSG00000198830
**Database:microRNA Target Name:hsa_ACCAAAG,MIR-9 ID:DB_ID:809**					
C=493; O=3; E=0.25; R=11.93; rawP=0.0019; adjP=0.0019					
Index	UserID	Gene Symbol	Gene Name	EntrezGene	Ensembl
1	*KITLG*	*KITLG*	KIT ligand	4254	ENSG00000049130
2	*CCDC43*	*CCDC43*	coiled-coil domain containing 43	124808	ENSG00000180329
3	*COL12A1*	*COL12A1*	collagen, type XII, alpha 1	1303	ENSG00000111799

Table 4 Enrichment for micro RNA 449 (miR449) related genes for all the methylation clusters (denoted by color).

Methylation cluster	Number of miR449 related genes	Number of not miR449 related genes	Percentage of miR449 genes present	Odds Ratio	Fisher p value	FDR corrected p value
All	268	19547	100			
black	109	6370	1.682358	1.245691	0.061912	0.130342
blue	191	11991	1.567887	1.153744	0.134	0.243636
brown	186	11605	1.577474	1.153496	0.143219	0.249077
cyan	82	3891	2.063932	1.536893	**0.001115***	**0.008338***
darkgreen	20	703	2.766252	2.074587	**0.005235***	**0.021351***
darkgrey	15	820	1.796407	1.333978	0.284564	0.392502
darkmagenta	6	360	1.639344	1.215595	0.643431	0.756978
darkolivegreen	4	319	1.23839	0.914569	1	1
darkorange	4	462	0.858369	0.631463	0.537006	0.683596
darkred	21	995	2.066929	1.539304	0.071587	0.143174
darkturquoise	19	749	2.473958	1.850108	**0.016935***	0.060097
green	197	10399	1.859192	1.38066	**0.000835***	**0.008338***
greenyellow	95	4636	2.008032	1.493732	**0.001251***	**0.008338***
grey	198	11512	1.690863	1.251039	**0.018029***	0.060097
grey60	73	4013	1.786588	1.326762	**0.035661***	0.089153
lightcyan	62	2984	2.035456	1.515091	**0.005338***	**0.021351***
lightgreen	22	958	2.244898	1.674892	**0.025257***	0.07355
lightyellow	14	1006	1.372549	1.014867	0.889579	0.961707
magenta	72	4019	1.759961	1.306167	0.050033	0.117724
midnightblue	81	4393	1.81046	1.344817	**0.025743***	0.07355
orange	6	436	1.357466	1.003714	1	1
orangered4	1	57	1.724138	1.279568	0.546877	0.683596
paleturquoise	4	356	1.111111	0.819519	1	1
pink	87	5337	1.603982	1.187151	0.171955	0.286591
plum1	3	105	2.777778	2.083792	0.182146	0.291433
purple	139	6852	1.988271	1.479495	**0.000265***	**0.006305***
red	175	9319	1.843269	1.369061	**0.001504***	**0.008593***
royalblue	26	977	2.592223	1.94093	**0.003444***	**0.017219***
saddlebrown	7	620	1.116427	0.823483	0.859114	0.954572
salmon	90	4195	2.10035	1.563072	**0.000473***	**0.006305***
sienna3	4	250	1.574803	1.166974	0.780178	0.891632
skyblue	10	638	1.54321	1.143197	0.605118	0.733477
skyblue3	3	112	2.608696	1.953568	0.206203	0.305485
steelblue	9	332	2.639296	1.977104	0.055395	0.123101
tan	103	5792	1.747243	1.296961	**0.0293***	0.078133
turquoise	180	12050	1.471791	1.078191	0.434365	0.579153
violet	1	294	0.338983	0.24807	0.194646	0.299456
white	15	697	2.106742	1.569591	0.099306	0.189154
yellow	219	11446	1.877411	1.394889	**0.000321***	**0.006305***
yellowgreen	5	234	2.09205	1.558406	0.264274	0.377535

*$p<0.05$ in a Fisher's exact test

Table 5 Enrichment for micro RNA 23A/23B (miR23A/miR23B) related genes for all the methylation clusters (denoted by color).

Methylation cluster	Number of miR23A/miR23B related genes	Number of not miR23A/miR23B related genes	Percentage of miR23A/miR23B genes present	Odds Ratio	Fisher p value	FDR corrected p value
All	388	18888	100			
Black	125	6354	1.92931	0.980542	0.876933	0.97437
Blue	275	11907	2.257429	1.145333	0.090175	0.150291
Brown	309	11482	2.620643	1.336477	**0.000194***	**0.000864***
Cyan	110	3863	2.768689	1.421835	**0.001925***	**0.005922***
Darkgreen	34	689	4.702628	2.463708	**1.11E-05***	**0.000111***
Darkgrey	27	808	3.233533	1.668413	**0.016055***	**0.033799***
darkmagenta	6	360	1.639344	0.832311	0.848498	0.97437
darkolivegreen	4	319	1.23839	0.626196	0.538432	0.694751
darkorange	13	453	2.7897	1.432982	0.234532	0.347454
Darkred	30	986	2.952756	1.519366	**0.037978***	0.066048
darkturquoise	36	732	4.6875	2.45566	**5.94E-06***	**9.78E-05***
Green	268	10328	2.529256	1.294863	**0.001427***	**0.004756***
greenyellow	124	4607	2.62101	1.343338	**0.005541***	**0.014776***
Grey	273	11437	2.331341	1.188753	**0.031269***	0.05956
grey60	104	3982	2.545277	1.304249	**0.021412***	**0.042825***
lightcyan	117	2929	3.841103	1.994367	**1.21E-09***	**4.85E-08***
lightgreen	31	949	3.163265	1.631224	**0.013915***	**0.030923***
lightyellow	19	1001	1.862745	0.947735	0.908	0.981622
magenta	121	3970	2.957712	1.521468	**0.000135***	**0.000755***
midnightblue	130	4344	2.905677	1.494441	**0.000151***	**0.000755***
orange	9	433	2.036199	1.037991	0.86174	0.97437
orangered4	3	55	5.172414	2.723566	0.106554	0.170487
paleturquoise	8	352	2.222222	1.134953	0.699199	0.847514
pink	122	5302	2.249263	1.147337	0.191923	0.295265
plum1	2	106	1.851852	0.942235	1	1
purple	189	6802	2.703476	1.387493	**0.000384***	**0.001534***
red	267	9227	2.812303	1.4444	**7.33E-06***	**9.78E-05***
royalblue	36	967	3.589232	1.859137	**0.001183***	**0.004301***
saddlebrown	15	612	2.392344	1.223963	0.463515	0.61802
salmon	106	4179	2.473746	1.265298	**0.037522***	0.066048
sienna3	5	249	1.968504	1.002777	1	1
skyblue	10	638	1.54321	0.782737	0.562594	0.703242
skyblue3	4	111	3.478261	1.799455	0.292754	0.41822
steelblue	14	327	4.105572	2.137922	**0.010054***	**0.025134***
tan	170	5725	2.8838	1.482782	**3.63E-05***	**0.000242***
turquoise	241	11989	1.970564	0.99597	1	1
violet	8	287	2.711864	1.391961	0.392259	0.541046
white	24	688	3.370787	1.74197	**0.013647***	**0.030923***
yellow	315	11350	2.700386	1.385325	**2.77E-05***	**0.000222***
yellowgreen	12	227	5.020921	2.639601	**0.003268***	**0.009338***

*p<0.05 in a Fisher's exact test

Table 6 Enrichment for micro RNA 9 (miR9) related genes for all the methylation clusters (denoted by color).

Methylation cluster	Number of miR9 related genes	Number of not miR9 related genes	Percentage of miR9 genes present	Odds Ratio	Fisher p value	FDR corrected p value
All	471	18805	100			
Black	168	6311	2.592993	1.091209	0.352824	0.431453
Blue	346	11836	2.840256	1.1922	**0.014562***	**0.03077***
Brown	356	11435	3.019252	1.266976	**0.001035***	**0.003764***
Cyan	129	3844	3.246917	1.378097	**0.001885***	**0.0058***
darkgreen	23	700	3.181189	1.349202	0.172396	0.246279
darkgrey	32	803	3.832335	1.636352	**0.011275***	**0.028189***
darkmagenta	16	350	4.371585	1.877447	**0.023084***	**0.046167***
darkolivegreen	12	311	3.71517	1.584633	0.137311	0.211248
darkorange	15	451	3.218884	1.365857	0.220659	0.304357
Darkred	35	981	3.444882	1.465245	**0.036233***	0.065878
darkturquoise	28	740	3.645833	1.553862	**0.030762***	0.058594
Green	341	10255	3.218196	1.364611	**2.03E-05***	**0.000247***
greenyellow	148	4583	3.128303	1.325515	**0.003827***	**0.010935***
Grey	348	11362	2.971819	1.254436	**0.001833***	**0.0058***
grey60	135	3951	3.303965	1.403281	**0.000844***	**0.003375***
lightcyan	120	2926	3.939593	1.683951	**2.02E-06***	**8.1E-05***
lightgreen	38	942	3.877551	1.656696	**0.00554***	**0.014773***
lightyellow	21	999	2.058824	0.863202	0.596849	0.663166
magenta	125	3966	3.055488	1.293958	**0.013142***	**0.03077***
midnightblue	135	4339	3.017434	1.277805	**0.014616***	**0.03077***
orange	14	428	3.167421	1.343389	0.269089	0.358785
orangered4	1	57	1.724138	0.720537	1	1
paleturquoise	11	349	3.055556	1.294451	0.381217	0.448491
pink	153	5271	2.820796	1.192889	**0.067614***	0.11269
plum1	2	106	1.851852	0.774915	1	1
purple	223	6768	3.189815	1.353138	**0.000322***	**0.001609***
red	301	9193	3.170423	1.344136	**9.54E-05***	**0.000636***
royalblue	34	969	3.389831	1.44101	**0.045612***	**0.079326***
saddlebrown	14	613	2.232855	0.937981	1	1
salmon	152	4133	3.547258	1.508769	**2.62E-05***	**0.000247***
sienna3	3	251	1.181102	0.490888	0.295266	0.380989
skyblue	21	627	3.240741	1.375513	0.151841	0.224949
skyblue3	4	111	3.478261	1.479937	0.355948	0.431453
steelblue	9	332	2.639296	1.113338	0.718295	0.776535
tan	194	5701	3.290925	1.397485	**0.00015***	**0.000859***
turquoise	313	11917	2.55928	1.067382	0.39254	0.448617
violet	12	283	4.067797	1.741421	0.079458	0.127133
white	37	675	5.196629	2.25118	**2.19E-05***	**0.000247***
yellow	370	11295	3.171882	1.344763	**3.08E-05***	**0.000247***
yellowgreen	15	224	6.276151	2.749983	**0.000769***	**0.003375***

*p<0.05 in a Fisher's exact test

Supplementary Note 1: Possible confounders in the blood replication sample

First the potential confounding by cellcount on average DNA methylation was examined (analysis 1 on p18).
Next we investigated the potential influence of current MDD and ethnicity per outcome variable (cg27512205 methylation levels, cortisol stress reactivity and childhood trauma)(analyses 2-7 on p19-20).
Moreover stratified analyses for current MDD and ethnicity were performed to investigate potential confounding (analyses 8-19 on p21-25).
Finally, to establish the influence of current MDD and ethnicity, they were added as covariates to the model examining the association between *KITLG* and cortisol stress reactivity (analysis 20 on p26).

First the potential confounding by cellcount on average DNA methylation in the blood replication sample is examined.

Aim analysis 1: Association between cellcount and average DNA methylation in the blood replication sample is examined.

Linear regression model 1: Average methylation ~ CD8 T cells + CD4 T cells + Natural Killer cells + Bcells + Monocytes + Granulocytes

Result 1 Average methylation ~Cellcount

	Estimate	*Std. Error*	*t value*	*Pr(>|t|)*
M$CD8	-4.629	1.126	-4.111	0.0000465713987 ***
M$CD4T	-4.306	1.146	-3.757	0.000194 ***
M$NK	-4.330	1.142	-3.792	0.000169 ***
M$Bcell	-3.804	1.047	-3.634	0.000310 ***
M$Mon	-3.731	1.233	-3.026	0.002618 **
M$Gran	-4.359	1.127	-3.867	0.000126 ***

Signif. codes: 0 ‘***’ 0.001 ‘**’ 0.01 ‘*’ 0.05 ‘.’ 0.1 ‘ ’ 1
R-squared: 0.02882, F-statistic: 3.335 on 6 and 466 DF, p-value: 0.003164

Conclusion 1: Different cellcounts are associated with average DNA methylation levels in the blood replication sample.

Next we investigated the association of ethnicity with our main variables (cg27512205 methylation levels, cortisol stress reactivity and childhood trauma) in the blood replication sample.

Aim analysis 2: Influence ethnicity on cg27512205 methylation levels in the blood replication sample.
Linear regression model 2: cg27512205 methylation ~ Race

Result 2 cg27512205 ~ Race

	Estimate	*Std. Error*	*t value*	*Pr(>|t|)*
RaceCaucasian	0.003545	0.052041	0.068	0.946
RaceOther	-0.065441	0.080285	-0.815	0.420

Signif. codes: 0 '***' 0.001 '**' 0.01 '*' 0.05 '.' 0.1 ' ' 1
R-squared: -0.02907, F-statistic: 0.3785 on 2 and 42 DF, p-value: 0.6872

Conclusion 2: Ethnicity is not significantly associated with cg27512205 methylation.

Aim analysis 3: Influence ethnicity on cortisol stress reactivity in the blood replication sample.
Linear regression model 3: Cortisol stress reactivity (AUCi) ~ Race

Result 3 AUCi ~ Race

	Estimate	*Std. Error*	*t value*	*Pr(>|t|)*
RaceCaucasian	331.61	116.31	2.851	0.00673 **
RaceOther	538.70	179.43	3.002	0.00450 **

Signif. codes: 0 '***' 0.001 '**' 0.01 '*' 0.05 '.' 0.1 ' ' 1
R-squared: 0.2064 F-statistic: 6.723 on 2 and 42 DF, p-value: 0.00293

Conclusion 3: Ethnicity is associated with cortisol stress reactivity, specifically lower cortisol stress reactivity levels in Afro-American compared to Caucasian individuals.

Aim analysis 4: Influence ethnicity on childhood trauma levels in the blood replication sample.
Linear regression model4: Childhood trauma (CTQ) ~ Race

Result 4 CTQ ~ Race

	Estimate	*Std. Error*	*t value*	*Pr(>|t|)*
RaceCaucasian	-1.847	8.298	-0.223	0.825
RaceOther	-7.635	12.802	-0.596	0.554

Signif. codes: 0 '***' 0.001 '**' 0.01 '*' 0.05 '.' 0.1 ' ' 1
R-squared: -0.03873, F-statistic: 0.1797 on 2 and 42 DF, p-value: 0.8362

Conclusion 4: Ethnicity is not significantly associated with different childhood trauma levels.

We also examined the association of current MDD with our main variables (cg27512205 methylation levels, cortisol stress reactivity and childhood trauma) in the blood replication sample.

Aim analysis 5: Influence current MDD on cg27512205 methylation levels in the blood replication sample.
Linear regression model 5: cg27512205 methylation ~ Current MDD

Result 5 cg27512205 ~ Current MDD

	Estimate	*Std. Error*	*t value*	*Pr(>|t|)*
MDDTRUE	-0.00432	0.05627	-0.077	0.939

Signif. codes: 0 '***' 0.001 '**' 0.01 '*' 0.05 '.' 0.1 ' ' 1
R-squared: -0.02312 F-statistic: 0.005893 on 1 and 43 DF, p-value: 0.9392

Conclusion 5: Current MDD is not significantly associated with cg27512205 methylation.

Aim analysis 6: Influence current MDD on cortisol stress reactivity in the blood replication sample.
Linear regression model 6: Cortisol stress reactivity (AUCi) ~ Current MDD

Result 6 AUCi ~ Current MDD

	Estimate	*Std. Error*	*t value*	*Pr(>|t|)*
MDDTRUE	-19.45	143.20	-0.136	0.893

Signif. codes: 0 '***' 0.001 '**' 0.01 '*' 0.05 '.' 0.1 ' ' 1
R-squared: -0.02282 F-statistic: 0.01844 on 1 and 43 DF, p-value: 0.8926

Conclusion 6: Current MDD is not significantly associated with cortisol stress reactivity.

Aim analysis 7: Influence current MDD on childhood trauma levels in the blood replication sample.
Linear regression model 7: Childhood trauma (CTQ) ~ Current MDD

Result 7 CTQ ~ Current MDD

	Estimate	*Std. Error*	*t value*	*Pr(>|t|)*
MDDTRUE	26.497	7.966	3.326	0.00181 **

Signif. codes: 0 '***' 0.001 '**' 0.01 '*' 0.05 '.' 0.1 ' ' 1
R-squared: 0.1862 F-statistic: 11.06 on 1 and 43 DF, p-value: 0.001808

Conclusion 7: Current MDD is associated with higher childhood trauma levels.

Stratified analyses for ethnicity were performed to investigate potential confounding in the blood replication sample.

Aim analysis 8: Examine the relationship between childhood trauma and cortisol stress reactivity in the Caucasian subset (N=17).

Linear regression model 8: Cortisol stress reactivity (AUCi) ~ Childhood trauma (CTQ) + Gender + Age

Result 8 AUCi ~CTQ in Caucasian individuals(N=17)

	Estimate	*Std. Error*	*t value*	*Pr(>|t|)*
CTQtotal	-7.920	2.168	-3.653	0.002920 **
GenderMale	134.828	148.758	0.906	0.381239
age	-6.458	13.245	-0.488	0.633960

Signif. codes: 0 '***' 0.001 '**' 0.01 '*' 0.05 '.' 0.1 ' ' 1

R-squared: 0.4158 F-statistic: 4.795 on 3 and 13 DF, p-value: 0.01836

Conclusion 8: In the Caucasian individuals(N=17) childhood trauma is associated with blunted cortisol stress reactivity.

Aim analysis 9: Examine the relationship between childhood trauma and cg27512205 methylation in the Caucasian subset (N=17).

Linear regression model 9: cg27512205 methylation ~ Childhood trauma (CTQ) + Gender + Age

Result 9 cg27512205 ~CTQ in Caucasian individuals(N=17)

	Estimate	*Std. Error*	*t value*	*Pr(>|t|)*
CTQtotal	0.0022236	0.0007355	3.023	0.00979 **
GenderMale	0.1242792	0.0504679	2.463	0.02853 *
age	-0.0093013	0.0044935	-2.070	0.05893 .

Signif. codes: 0 '***' 0.001 '**' 0.01 '*' 0.05 '.' 0.1 ' ' 1

R-squared: 0.5051 F-statistic: 6.444 on 3 and 13 DF, p-value: 0.006569

Conclusion 9: In the Caucasian individuals(N=17) childhood trauma is associated with cg27512205 methylation.

Aim analysis 10: Examine the relationship between cg27512205 methylation and cortisol stress reactivity in the Caucasian subset (N=17).

Linear regression model 10: Cortisol stress reactivity (AUCi) ~cg27512205 methylation + Gender + Age

Result 10 AUCi ~ cg27512205 in Caucasian individuals(N=17)

	Estimate	*Std. Error*	*t value*	*Pr(>|t|)*
cg27512205	-1960.96	706.75	-2.775	0.01578 *
GenderMale	386.03	187.84	2.055	0.06054 .
age	-22.26	16.70	-1.332	0.20562

Signif. codes: 0 ‘***’ 0.001 ‘**’ 0.01 ‘*’ 0.05 ‘.’ 0.1 ‘ ’ 1

R-squared: 0.2564 F-statistic: 2.839 on 3 and 13 DF, p-value: 0.07911

Conclusion 10: In the Caucasian individuals(N=17) cg27512205 methylation is associated with blunted cortisol stress reactivity.

Aim analysis 11: Examine the relationship between childhood trauma and cortisol stress reactivity in the Afro-American subset (N=23).

Linear regression model 11: Cortisol stress reactivity (AUCi) ~ Childhood trauma (CTQ) + Gender + Age

Result 11 AUCi ~CTQ in Afro-American individuals(N=23)

	Estimate	*Std. Error*	*t value*	*Pr(>|t|)*
CTQtotal	3.075	4.021	0.765	0.4538
GenderMale	395.486	213.005	1.857	0.0789 .
age	-5.913	9.767	-0.605	0.5521

Signif. codes: 0 '***' 0.001 '**' 0.01 '*' 0.05 '.' 0.1 ' ' 1
R-squared: 0.02171 F-statistic: 1.163 on 3 and 19 DF, p-value: 0.3499

Conclusion 11: In the Afro-American individuals(N=23) childhood trauma is not significantly associated with blunted cortisol stress reactivity.

Aim analysis 12: Examine the relationship between childhood trauma and cg27512205 methylation in the Afro-American subset (N=23).

Linear regression model 12: cg27512205 methylation ~ Childhood trauma (CTQ) + Gender + Age

Result 12 cg27512205 ~CTQ in Afro-American individuals(N=23)

	Estimate	*Std. Error*	*t value*	*Pr(>|t|)*
CTQtotal	-0.001558	0.001794	-0.868	0.396
GenderMale	-0.195203	0.095030	-2.054	0.054 .
age	0.004530	0.004357	1.039	0.312

Signif. codes: 0 '***' 0.001 '**' 0.01 '*' 0.05 '.' 0.1 ' ' 1
R-squared: 0.06702 F-statistic: 1.527 on 3 and 19 DF, p-value: 0.2399

Conclusion 12: In the Afro-American individuals(N=23) childhood trauma is not significantly associated with cg27512205 methylation.

Aim analysis 13: Examine the relationship between cg27512205 methylation and cortisol stress reactivity in the Afro-American subset (N=23).

Linear regression model 13: Cortisol stress reactivity (AUCi) ~cg27512205 methylation + Gender + Age

Result 13 AUCi ~ cg27512205 in Afro-American individuals(N=23)

	Estimate	*Std. Error*	*t value*	*Pr(>|t|)*
cg27512205	-745.182	482.636	-1.544	0.13908
GenderMale	210.845	203.878	1.034	0.31404
age	-2.139	9.531	-0.224	0.82483

Signif. codes: 0 '***' 0.001 '**' 0.01 '*' 0.05 '.' 0.1 ' ' 1
R-squared: 0.104 F-statistic: 1.851 on 3 and 19 DF, p-value: 0.1722

Conclusion 13: In the Afro-American individuals(N=23) cg27512205 methylation is not significantly associated with cortisol stress reactivity.

<u>Stratified analyses for current MDD were performed to investigate potential confounding in the blood replication sample.</u>

Aim analysis 14: Examine the relationship between childhood trauma and cortisol stress reactivity in the individuals without current MDD(N=34).

Linear regression model 14: Cortisol stress reactivity (AUCi) ~ Childhood trauma (CTQ) + Gender + Age

Result 14 AUCi ~CTQ in current non-MDD individuals(N=34)

	Estimate	*Std. Error*	*t value*	*Pr(>|t|)*
CTQtotal	-4.528	2.799	-1.618	0.116
GenderMale	231.053	149.441	1.546	0.133
age	-7.924	8.941	-0.886	0.382

Signif. codes: 0 ‘***’ 0.001 ‘**’ 0.01 ‘*’ 0.05 ‘.’ 0.1 ‘ ’ 1
R-squared: 0.08806 F-statistic: 2.062 on 3 and 30 DF, p-value: 0.1263

Conclusion 14: In the individuals without current MDD(N=34) childhood trauma is not significantly associated with cortisol stress reactivity.

Aim analysis 15: Examine the relationship between childhood trauma and cg27512205 methylation in the individuals without current MDD(N=34).

Linear regression model 15: cg27512205 methylation ~ Childhood trauma (CTQ) + Gender + Age

Result 15 cg27512205 ~CTQ in current non-MDD individuals(N=34)

	Estimate	*Std. Error*	*t value*	*Pr(>|t|)*
CTQtotal	0.001782	0.001059	1.683	0.103
GenderMale	0.006236	0.056527	0.110	0.913
age	-0.001853	0.003382	-0.548	0.588

R-squared: -0.001786 F-statistic: 0.9804 on 3 and 30 DF, p-value: 0.4151

Conclusion 15: In the individuals without current MDD(N=34) childhood trauma is not significantly associated with cg27512205 methylation.

Aim analysis 16: Examine the relationship between cg27512205 methylation and cortisol stress reactivity in the individuals without current MDD(N=34).

Linear regression model 16: Cortisol stress reactivity (AUCi) ~cg27512205 methylation + Gender + Age

Result 16 AUCi ~ cg27512205 in current non-MDD individuals(N=34)

	Estimate	*Std. Error*	*t value*	*Pr(>|t|)*
cg27512205	-1014.161	444.028	-2.284	0.0296 *
GenderMale	244.389	143.654	1.701	0.0992 .
age	-10.946	8.544	-1.281	0.2099

Signif. codes: 0 ‘***’ 0.001 ‘**’ 0.01 ‘*’ 0.05 ‘.’ 0.1 ‘ ’ 1
R-squared: 0.1554 F-statistic: 3.023 on 3 and 30 DF, p-value: 0.04494

Conclusion 16: In the individuals without current MDD(N=34) cg27512205 methylation is associated with blunted cortisol stress reactivity.

Aim analysis 17: Examine the relationship between childhood trauma and cortisol stress reactivity in the individuals with current MDD(N=11). All female so no gender covariate.

Linear regression model 17: Cortisol stress reactivity (AUCi) ~ Childhood trauma (CTQ) + Age

Result 17 AUCi ~CTQ in current MDD individuals(N=11)				
	Estimate	*Std. Error*	*t value*	*Pr(>|t|)*
CTQtotal	-11.410	7.313	-1.560	0.1573
age	-27.607	33.108	-0.834	0.4286

Signif. codes: 0 '***' 0.001 '**' 0.01 '*' 0.05 '.' 0.1 ' ' 1				
Adjusted R-squared: 0.06998 F-statistic: 1.376 on 2 and 8 DF, p-value: 0.3064				

Conclusion 17: In the individuals with current MDD(N=34) childhood trauma is not significantly associated with cortisol stress reactivity.

Aim analysis 18: Examine the relationship between childhood trauma and cg27512205 methylation in the individuals with current MDD(N=11). All female so no gender covariate.
Linear regression model 18: cg27512205 methylation ~ Childhood trauma (CTQ) + Age

Result 18 cg27512205 ~CTQ in current MDD individuals(N=11)				
	Estimate	*Std. Error*	*t value*	*Pr(>|t|)*
CTQtotal	0.001441	0.002713	0.531	0.610
age	0.012779	0.012282	1.040	0.329
R-squared: -0.08629 F-statistic: 0.6028 on 2 and 8 DF, p-value: 0.5704				

Conclusion 18: In the individuals with current MDD(N=11) childhood trauma is not significantly associated with cg27512205 methylation.

Aim analysis 19: Examine the relationship between cg27512205 methylation and cortisol stress reactivity in the individuals with current MDD(N=11). All female so no gender covariate
Linear regression model 19: Cortisol stress reactivity (AUCi) ~cg27512205 methylation + Age

Result 19 AUCi ~ cg27512205 in current MDD individuals(N=11)				
	Estimate	*Std. Error*	*t value*	*Pr(>|t|)*
res_cg27512205	-1885.263	836.654	-2.253	0.0543 .
age	3.517	30.677	0.115	0.9116

Signif. codes: 0 '***' 0.001 '**' 0.01 '*' 0.05 '.' 0.1 ' ' 1				
R-squared: 0.258 F-statistic: 2.738 on 2 and 8 DF, p-value: 0.1242				

Conclusion 19: In the individuals with current MDD(N=11) cg27512205 methylation is not significantly associated with blunted cortisol stress reactivity.

To establish the influence of current MDD and ethnicity, they were added as covariates to the model examining the association between KITLG and cortisol stress reactivity in the blood replication sample.

Aim analysis 20: Influence current MDD and ethnicity on the model examining the association between cg27512205 methylation levels and cortisol stress reactivity in the blood replication sample.

Linear regression model 20: Cortisol stress reactivity (AUCi) ~ methylation cg27512205 + Gender + age + Race + MDD

Result 20 AUCi ~ cg27512205 in all individuals (N=45)

	Estimate	*Std. Error*	*t value*	*Pr(>|t|)*
cg27512205	-1039.583	358.600	-2.899	0.006187 **
GenderMale	213.211	134.351	1.587	0.120809
age	-1.501	7.980	-0.188	0.851794
RaceCaucasian	300.603	115.727	2.598	0.013285 *
RaceOther	453.413	176.659	2.567	0.014334 *
MDDTRUE	-3.643	122.702	-0.030	0.976467

Signif. codes: 0 ‘***’ 0.001 ‘**’ 0.01 ‘*’ 0.05 ‘.’ 0.1 ‘ ’ 1

R-squared: 0.3278 F-statistic: 4.576 on 6 and 38 DF, p-value: 0.001382

Conclusion 20: The association between cg27512205 methylation levels and cortisol stress reactivity in the blood replication sample remains present after adding ethnicity and current MDD as covariate. Ethnicity is associated with altered cortisol stress responses and an important determinant, therefore ethnicity was added as covariate to all analyses in the blood replication sample.

Supplementary Note 2: Influence of cell count in the discovery sample

The association of cellcount with cortisol stress reactivity (analysis 1 on p27), childhood trauma (analysis 2 p28), average methylation (analysis 3 on p29) and *KITLG* methylation (analysis 4 on p30) is examined. Finally, to establish the influence of cellcount on the association between *KITLG* and cortisol stress reactivity, we show the model summary for the entire model (analysis 5 on p31).

Aim analysis 1: Association between cellcount and cortisol stress reactivity (AUCi) in the discovery sample (N=85).
Linear regression model 1: Cortisol stress reactivity (AUCi) ~ CD8 T cells + Monocytes + Natural Killer cells + CD4 T cells + Bcells

Result 1 AUCi ~Cellcount

	Estimate	*Std. Error*	*t value*	*Pr(>|t|)*
CD8T	-415.8	5641.8	-0.074	0.941
Mono	106.0	2596.5	0.041	0.968
NK	733.9	1307.2	0.561	0.576
CD4T	-159.7	1257.7	-0.127	0.899
Bcell	-1570.0	2047.6	-0.767	0.445

R-squared: -0.04507 F-statistic: 0.2754 on 5 and 79 DF, p-value: 0.9253

Conclusion 1: Different cellcounts are not significantly associated with cortisol stress reactivity (AUCi) in the discovery sample.

Aim analysis 2: Association between cellcount and childhood trauma in the discovery sample (N=85).

Linear regression model 2: Childhood trauma(CTQ) ~ CD8 T cells + Monocytes + Natural Killer cells + CD4 T cells + Bcells

Result 2 CTQ ~Cellcount

	Estimate	*Std. Error*	*t value*	*Pr(>|t|)*
CD8T	-13.84346	107.14982	-0.129	0.89753
Mono	- 0.06564	49.31389	-0.001	0.99894
NK	19.01293	24.82724	0.766	0.44607
CD4T	44.29173	23.88644	1.854	0.06743 .
Bcell	33.45139	38.88764	0.860	0.39228

Signif. codes: 0 ‘***’ 0.001 ‘**’ 0.01 ‘*’ 0.05 ‘.’ 0.1 ‘ ’ 1

R-squared: 0.01953 F-statistic: 1.335 on 5 and 79 DF, p-value: 0.2583

Conclusion 2: Different cellcounts are not significantly associated with childhood trauma in the discovery sample.

Aim analysis 3: Association between cellcount and average DNA methylation in discovery sample (N=85).
Linear regression model 3: Average methylation (PC1) ~ CD8 T cells + Monocytes + Natural Killer cells + CD4 T cells + Bcells

Result 3 Average methylation ~Cellcount

	Estimate	*Std. Error*	*t value*	*Pr(>|t|)*
CD8T	-0.018781	0.035078	-0.535	0.59387
Mono	0.010193	0.016144	0.631	0.52963
NK	0.021518	0.008128	2.648	0.00979 **
CD4T	0.016304	0.007820	2.085	0.04031 *
Bcell	0.006590	0.012731	0.518	0.60617

Signif. codes: 0 ‘***’ 0.001 ‘**’ 0.01 ‘*’ 0.05 ‘.’ 0.1 ‘ ’ 1
R-squared: 0.06489 F-statistic: 2.166 on 5 and 79 DF, p-value: 0.06622

Conclusion 3: Different cellcounts are significantly associated with average DNA methylation levels in the discovery sample.

Aim analysis 4: Association between cellcount and cg27512205 DNA methylation in discovery sample (N=85).

Linear regression model 4: cg27512205 methylation ~ CD8 T cells + Monocytes + Natural Killer cells + CD4 T cells + Bcells

Result 4 cg27512205 ~Cellcount

	Estimate	*Std. Error*	*t value*	*Pr(>|t|)*
CD8T	-2.21662	2.27124	-0.976	0.332
Mono	1.12029	1.04530	1.072	0.287
NK	-0.05001	0.52626	-0.095	0.925
CD4T	-0.22508	0.50632	-0.445	0.658
Bcell	0.53399	0.82430	0.648	0.519

Signif. codes: 0 '***' 0.001 '**' 0.01 '*' 0.05 '.' 0.1 ' ' 1
R-squared: -0.007898 F-statistic: 0.8684 on 5 and 79 DF, p-value: 0.5063

Conclusion 4: Different cellcounts are not significantly associated with cg27512205 DNA methylation levels in the discovery sample.

Aim analysis 5: Association between cortisol stress reactivity and cg27512205 DNA methylation when cellcounts are included as covariates in discovery sample (N=85).

Linear regression model 5: Cortisol stress reactivity (AUCi) ~ cg27512205 methylation +Gender + age + CD8 T cells + Monocytes + Natural Killer cells + CD4 T cells + Bcells

Result 5 AUCi ~ cg27512205 +gender+age+ cellcount

	Estimate	*Std. Error*	*t value*	*Pr(>|t|)*
cg27512205	-1124.268	228.417	-4.922	4.85e-06 ***
GenderMale	-327.472	78.776	-4.157	8.38e-05 ***
age	3.502	2.570	1.363	0.177059
CD8T	845.056	4700.716	0.180	0.857810
CD4T	-108.060	1066.305	-0.101	0.919547
NK	508.869	1072.352	0.475	0.636479
Bcell	273.679	1709.032	0.160	0.873198
Mono	1819.355	2124.956	0.856	0.394587

Signif. codes: 0 ‘***’ 0.001 ‘**’ 0.01 ‘*’ 0.05 ‘.’ 0.1 ‘ ’ 1

R-squared: 0.3123 F-statistic: 5.768 on 8 and 76 DF, p-value: 9.672e-06

Conclusion 5: The association between cortisol stress reactivity and cg27512205 DNA methylation levels remains when cellcounts are added as covariates in the discovery sample.

Supplementary Note 3: Summary of the KITLG model in the cross-tissue replication sample

Aim analysis 1: Association between cg27512205 DNA methylation and cortisol stress reactivity in the cross-tissue replication sample (N=255).

Linear regression model 1: Cortisol stress reactivity (AUCi) ~ cg27512205 methylation +Gender + age

Result 1 AUCi ~ cg27512205 +gender+age

	Estimate	*Std. Error*	*t value*	*Pr(>|t|)*
cg27512205	-104.038	35.373	-2.941	0.00358 **
age	-4.835	13.871	-0.349	0.72771
GenderMale	11.691	11.664	1.002	0.31716

Signif. codes: 0 '***' 0.001 '**' 0.01 '*' 0.05 '.' 0.1 ' ' 1
R-squared: 0.02855 F-statistic: 3.489 on 3 and 251 DF, p-value: 0.01638

Conclusion 1: Cg27512205 DNA methylation levels are associated with cortisol stress reactivity in the cross-tissue sample.

Supplementary References

1. Barfield,R.T. *et al.* Accounting for population stratification in DNA methylation studies. *Genet. Epidemiol.* **38**, 231-241 (2014).

2. Carpenter,L.L. *et al.* Decreased adrenocorticotropic hormone and cortisol responses to stress in healthy adults reporting significant childhood maltreatment. *Biol. Psychiatry* **62**, 1080-1087 (2007).

3. Carpenter,L.L., Shattuck,T.T., Tyrka,A.R., Geracioti,T.D., & Price,L.H. Effect of childhood physical abuse on cortisol stress response. *Psychopharmacology (Berl)* **214**, 367-375 (2011).

4. Elzinga,B.M. *et al.* Diminished cortisol responses to psychosocial stress associated with lifetime adverse events a study among healthy young subjects. *Psychoneuroendocrinology* **33**, 227-237 (2008).

5. Heim,C. *et al.* Pituitary-adrenal and autonomic responses to stress in women after sexual and physical abuse in childhood. *JAMA* **284**, 592-597 (2000).

6. Moran-Santa Maria,M.M. *et al.* Influence of cocaine dependence and early life stress on pituitary-adrenal axis responses to CRH and the Trier social stressor. *Psychoneuroendocrinology* **35**, 1492-1500 (2010).

7. Pesonen,A.K. *et al.* Childhood separation experience predicts HPA axis hormonal responses in late adulthood: a natural experiment of World War II. *Psychoneuroendocrinology* **35**, 758-767 (2010).

8. Witt,S.H. *et al.* An interaction between a neuropeptide Y gene polymorphism and early adversity modulates endocrine stress responses. *Psychoneuroendocrinology* **36**, 1010-1020 (2011).

9. Buchmann,A.F. *et al.* Moderating role of FKBP5 genotype in the impact of childhood adversity on cortisol stress response during adulthood. *Eur. Neuropsychopharmacol.* **24**, 837-845 (2014).

10. Kraft,A.J. & Luecken,L.J. Childhood parental divorce and cortisol in young adulthood: evidence for mediation by family income. *Psychoneuroendocrinology* **34**, 1363-1369 (2009).

11. Luecken,L.J. Childhood attachment and loss experiences affect adult cardiovascular and cortisol function. *Psychosom. Med.* **60**, 765-772 (1998).

12. Otte,C. *et al.* Association between childhood trauma and catecholamine response to psychological stress in police academy recruits. *Biol. Psychiatry* **57**, 27-32 (2005).

13. Goldman-Mellor,S., Hamer,M., & Steptoe,A. Early-life stress and recurrent psychological distress over the lifecourse predict divergent cortisol reactivity patterns in adulthood. *Psychoneuroendocrinology* **37**, 1755-1768 (2012).

14. Elzinga,B.M., Spinhoven,P., Berretty,E., de,J.P., & Roelofs,K. The role of childhood abuse in HPA-axis reactivity in Social Anxiety Disorder: a pilot study. *Biol. Psychol.* **83**, 1-6 (2010).

15. Videlock,E.J. *et al.* Childhood trauma is associated with hypothalamic-pituitary-adrenal axis responsiveness in irritable bowel syndrome. *Gastroenterology* **137**, 1954-1962 (2009).

16. Bremner,J.D. *et al*. Cortisol response to a cognitive stress challenge in posttraumatic stress disorder (PTSD) related to childhood abuse. *Psychoneuroendocrinology* **28**, 733-750 (2003).

17. Santa Ana,E.J. *et al*. PTSD and the HPA axis: differences in response to the cold pressor task among individuals with child vs. adult trauma. *Psychoneuroendocrinology* **31**, 501-509 (2006).
